# Subclinical ketosis in postpartum dairy cows alters the adipose tissue immunological profile in a depot-specific manner

**DOI:** 10.3389/fimmu.2025.1578669

**Published:** 2025-06-17

**Authors:** Tainara C. Michelotti, Bruno C. Menarim, Alexandra P. Tegeler, Jean F. Fiallo Diez, Vinicius S. Machado, Yava Jones-Hall, Oscar J. Benitez, Shavahn C. Loux, Clarissa Strieder-Barboza

**Affiliations:** ^1^ Department of Veterinary Sciences, Davis College of Agricultural Sciences and Natural Resources, Texas Tech University, Lubbock, TX, United States; ^2^ Gluck Equine Research Center, Department of Veterinary Sciences, Martin-Gatton College of Agricultural, Food and Environment, University of Kentucky, Lexington, KY, United States; ^3^ School of Veterinary Medicine & Biomedical Sciences, Texas A&M, College Station, TX, United States; ^4^ School of Veterinary Medicine, Texas Tech University, Amarillo, TX, United States; ^5^ Department of Animal Sciences, College of Agriculture, Louisiana State University, Baton Rouge, LA, United States

**Keywords:** transcriptome, ketosis, visceral adipose tissue, subcutaneous adipose tissue, immune response

## Abstract

**Introduction:**

Subclinical ketosis (SCK) is a common metabolic disorder linked to adipose tissue (AT) dysfunction in periparturient dairy cows. While subcutaneous AT (SAT) and visceral AT (VAT) differ in structure, cellularity, and function, depot-specific responses to ketosis remain poorly understood. This study aimed to determine the transcriptional differences of flank SAT and omental VAT in early lactation dairy cows in response to SCK.

**Methods:**

Multiparous Holstein dairy cows within the first 10 days postpartum were screened for SCK. Subclinical ketosis was defined as blood β-hydroxybutyrate (BHB) concentrations between 1.4 and 2.6 mmol/L, while control, non-ketotic animals (NK) had BHB equal to or lower than 0.8 mmol/L. Adipose tissue biopsies were obtained from flank SAT and omental VAT from five SCK and five NK cows for RNA sequencing and immunohistochemistry analyses.

**Results and Discussion:**

Subclinical ketosis affected AT transcriptional profiles in a depot-specific manner. In SAT, transcriptional changes related to SCK reflected homeostatic AT remodeling and immune cell infiltration indicative of inflammatory responses, fibroplasia, and the negative regulation of adaptive immunity responses. In VAT, SCK-related transcriptional changes reflected increased pro-inflammatory responses linked to impaired lipid metabolism and dysregulation of focal adhesion and endocytosis. Tissue expression of proteins coded by genes differentially expressed between SCK and NK revealed a depot-dependent response on AT, indicating a higher infiltration of macrophages and B cells in SCK cows. Overall, our study provides new insights into molecular mechanisms underlying ketosis pathogenesis, highlighting the dysregulation of inflammatory responses, lipid metabolism, and insulin signaling in both SAT and VAT of postpartum dairy cows.

## Introduction

The transition from gestation to lactation involves drastic changes on endocrine and energy metabolism and, hence, nutritional demands (1–3). Periparturient dairy cows face a concomitant reduction on feed intake and an increase in energy demand, thus generating a negative energy balance (NEB) (4, 5). Increased lipolysis and insulin resistance in peripheral adipose tissue (AT) are major adaptive mechanisms to cope with NEB and to prioritize glucose use for milk synthesis (6, 7). Therefore, higher circulating concentrations of alternate energy sources, such as non-esterified fatty acids (NEFA) and β-hydroxybutyrate (BHB), are expected during this stage. However, we and others have demonstrated that, in periparturient cows, uncontrolled lipolysis during NEB leads to dysfunctional AT responses such as dysregulated inflammation and persistent insulin resistance (8–11), and increased risk of metabolic disorders, including ketosis (12, 13).

Ketosis is a metabolic disorder resulting from poor adaptive responses to NEB due to an overwhelming amount of NEFA being metabolized through ketogenesis in the liver. Besides hyperketonemia, clinical ketosis manifests as a loss of appetite, decreased milk production, and rapid loss of body condition (14). On the other hand, subclinical ketosis (SCK) is a condition with less evident clinical signs defined by serum BHB concentrations ≥ 1.4 mmol/L (15, 16). SCK affects 40% to 60% of early lactation dairy cows (17) and leads to significant losses in herd profitability associated with decreased milk production and reproductive performance, and increased risk of other health disorders (18, 19). While dysfunctional AT responses to NEB are a triggering factor for ketosis, how SCK affects depot-specific AT transcriptional profiles in postpartum dairy cows remains poorly understood and limits the development of targeted preventive interventions.

AT supports whole-body metabolic function and energy demand of early lactation cows in a depot-specific manner. For example, retroperitoneal visceral AT (VAT) is more lipolytically active (20) and maintains higher insulin sensitivity than tailhead subcutaneous AT (SAT) in periparturient dairy cows (21), indicating that VAT may play a greater role in providing energy substrates during early lactation. Recently, using single-nucleus RNA sequencing, we revealed an enrichment of adipose stromal and progenitor cells and endothelial cells in SAT from the flank, and an increased diversity and abundance of macrophages, natural killer cells, and T cells in omental VAT (22). These findings suggested higher adipogenic and angiogenic potentials of SAT, and a pro-inflammatory profile of VAT (22). Notably, features of AT metabolic dysregulation also depend on its anatomical location. For instance, omental VAT of periparturient dairy cows with SCK has an increased inflammatory response and infiltration of macrophages compared with SAT from tailhead and other VAT depots (mesenteric, retroperitoneal, and perirenal) (23). Altogether, these findings suggest a more beneficial metabolic role of SAT, while VAT undergoes more dynamic changes and may contribute more to metabolic disorder development. We hypothesize that SCK differently affects SAT and VAT transcriptome as these depots differ in structure, cellularity, and metabolic function. By elucidating the impact of SCK on depot-specific transcriptome, we aim to unravel depot-specific mechanisms by which AT is involved in ketosis pathogenesis. Our objective was to determine transcriptional and immunohistochemical differences in the omental VAT and flank SAT of postpartum dairy cows with SCK.

## Materials and methods

### Experimental design

The Institutional Animal Care and Use Committee (IACUC) of the Texas Tech University approved all the procedures for this study (protocol no. 21024-04). The experiment was conducted from February to March 2022 at a commercial dairy farm located near Friona, TX. Experimental dairy cows were housed in a free-stall installation, fed a balanced total mixed ratio diet provided *ad libitum*, and milked twice daily in a rotary milking system.

**Figure 1:**
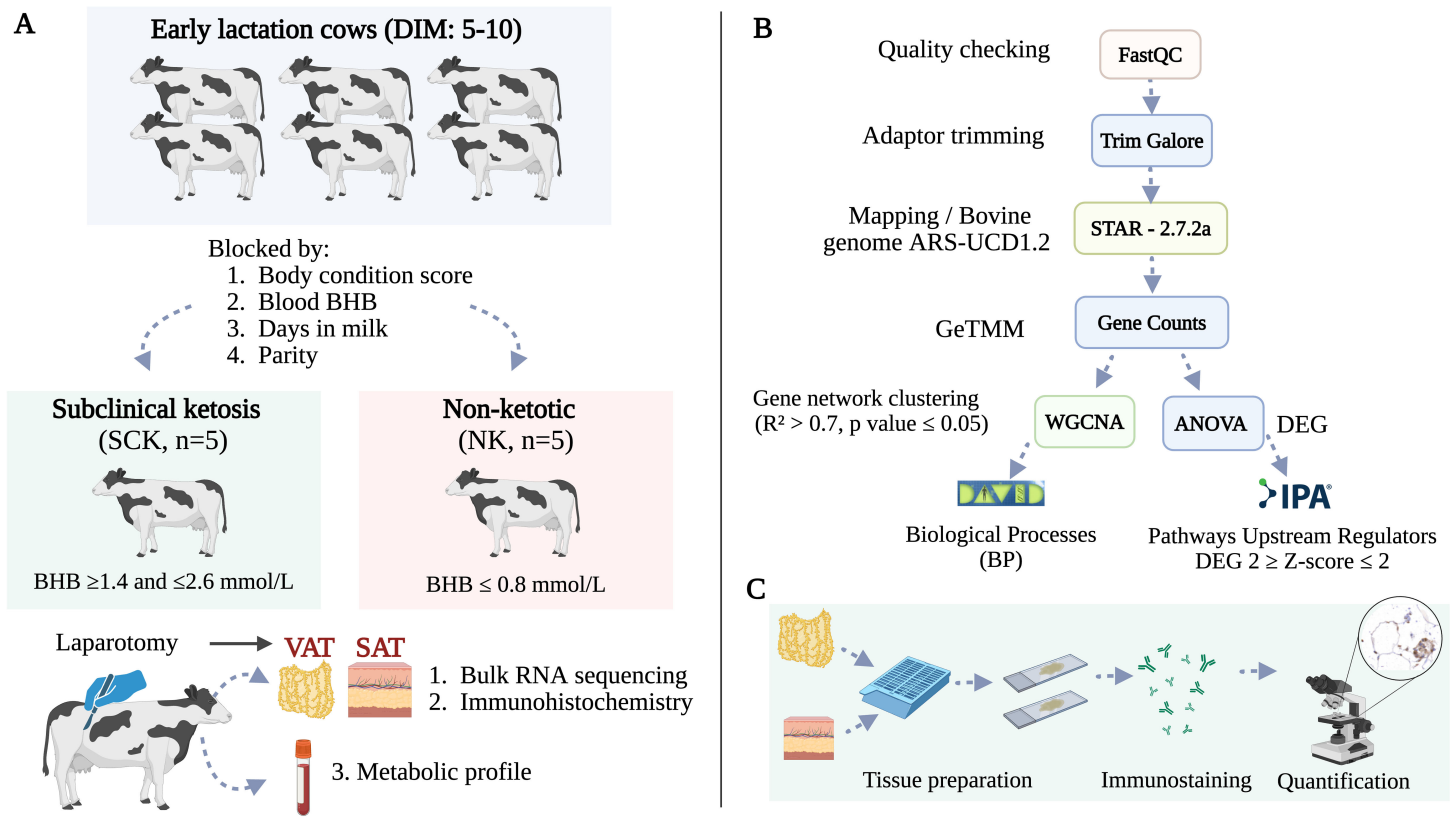
**(A)** Study design. Ten multiparous Holstein dairy cows in early lactation were enrolled in the study based on the diagnosis of subclinical ketosis (*n* = 5, BHB ≥ 1.4 and ≤ 2.6 mmol/L). Non-ketotic (NK, *n* = 5, BHB ≤ 0.8 mmol/L) cows were matched with SCK animals according to parity, days in milk, and body condition score. Blood samples for a metabolic profile and AT biopsies from flank subcutaneous AT (SAT) and omental visceral AT (VAT) were obtained for transcriptomics and immunohistochemistry at the time of ketosis diagnosis via right flank laparotomy. **(B)** Bioinformatics pipeline used for RNA sequencing, quantification, and analysis. **(C)** Immunohistochemistry analysis of visceral and subcutaneous AT immune markers. Created using BioRender.

The study design is summarized in **Figure 1A** and recently described by our group (24). Briefly, using a cross-sectional design, multiparous Holstein dairy cows within the first 10 days postpartum were screened for SCK based on blood concentrations of BHB determined by a Precision Xtra Blood Glucose and Ketone Monitoring System (Abbott Laboratories, Chicago, IL, USA). Five cows with SCK (*n* = 5, BHB ≥ 1.4 and ≤ 2.6 mmol/L) and five non-ketotic cows (NK, *n* = 5, BHB ≤ 0.8 mmol/L) were enrolled in the study. SCK and NK cows were matched by parity, body condition score, and days in milk. Experimental cows had an average body condition score of 3.6 ± 0.3 [1–5 scale (25)], 3.2 ± 1.4 lactations, and were 7.6 ± 1.9 days postpartum. Body condition score was evaluated by two experienced individuals. Records from each cow were checked for concurrent diseases and only animals without any other reported postpartum health problem were enrolled in the study. Descriptive statistics of parity, days in milk, BHB concentration, and body condition score in SCK and NK cows were assessed using a paired *t*-test of SAS 9.4 (SAS Institute Cary NC, USA) and results are found in **Table 1**. Sample size was defined per convenience based on previous AT transcriptome studies analyzing depot (22) and ketosis effects (26) in dairy cows.

**Table 1:** Comparison of SCK (subclinical ketosis) and NK (non-ketotic) cows.

Parameters	NK		SCK		*p*-value
	Mean	SD^1^	Mean	SD^1^	
Days in milk	7.4	1.8	7.8	2.0	0.59
Body condition score	3.7	0.2	3.6	0.4	0.55
Parity	3.2	1.6	3.2	1.2	1.00
BHB^2^, mmol/L	0.72	0.07	1.58	0.10	<0.01

^1^ Standard deviation.

^2^ β-hydroxybutyrate measured with Precision Xtra Blood Glucose and Ketone Monitoring System (Abbott Laboratories, Chicago, IL, USA).

### Blood collection and analyses

Blood samples were obtained from the coccygeal vessels before morning feeding (for ketosis diagnosis) and prior to AT collection (for metabolic profiling) using a 21-gauge vacutainer needle (BD Vacutainer, Becton Dickinson, Cat. No. 368650). Blood samples were collected into tubes (BD Vacutainer, Becton Dickinson, Cat. No. 367820) containing a serum clot activator. After collection, tubes were kept at 21°C for a maximum of 4 h until centrifugation. Serum was obtained by centrifugation at 1,300 × *g* for 15 min at 21°C and aliquots were frozen at −80°C until further analysis. Blood samples were analyzed for a metabolic profile including the concentrations of glucose, BHB, NEFA, blood urea nitrogen (BUN), albumin, cholesterol, sodium, calcium, phosphorus, potassium, and chloride determined on a Beckman Coulter AU 480 automated analyzer using commercially available reagents at the Texas A&M Veterinary Medical Diagnostic Laboratory (College Station, TX). Differences on concentrations of blood biomarkers were evaluated using the MIXED procedure of SAS 9.4 (SAS Institute Cary NC, USA). Results were log-scale transformed if needed to comply with normal distribution of residuals. The statistical model contained the effect of group (NK or SCK) as fixed effect, while the cow within group and block was considered as a random effect. Statistical significance was declared at *p*-value ≤ 0.05 and tendencies were declared at *p*-value ≤ 0.10.

### Adipose tissue collection

Matched flank SAT and omental VAT samples were obtained from each cow through right flank laparotomy, as reported by our group (24, 27). Surgeries were performed in paired SCK and NK cows every day of collection. Briefly, surgical site was prepared by clipping a large area (>30 cm) on the right flank to remove the hair followed by alternate scrubbing with 2% chlorhexidine and 70% alcohol (three times each). Local anesthesia was applied using 50 mL of 2% lidocaine hydrochloride (VetOne, Cat. No. 510213) in an inverted L block, at least 10 cm from the biopsy site. A 7- to 10-cm skin incision was made 10 cm caudally and parallel to the last rib, and 7–10 cm below the proximal margin of the paralumbar fossa. A 5- to 10-g sample of SAT was obtained from underneath the flank incision. Next, muscles of the abdominal wall were incised and dissected until reaching the peritoneum. A biopsy of approximately 10 g was obtained from the omental VAT. Muscle layers were closed with simple continuous suture pattern using polyglactin 910 - USP 3 (Riverpoint Medical, Portland, OR, USA), while skin incision was closed with Ford interlocking suture pattern using non-absorbable polyamide USP 3 (Braunamid, Cat. No. J009103, B. Braun Surgical, Rubi, Spain). Animals were clinically monitored in the next 3 days after surgery for pain, fever, and local inflammation, and treated with flunixin meglumine. Non-absorbable sutures were removed after 7–10 days. Immediately after harvest, SAT and VAT samples used for transcriptome analysis were snap-frozen and stored in liquid nitrogen until further analysis. For immunohistochemistry analysis, aliquots of the same samples were collected in cold 1× phosphate-buffered saline (PBS) followed by fixation in formalin, as described below.

### Transcriptome analysis of adipose tissue

#### RNA isolation and sequencing

Adipose tissue total RNA was extracted using Trizol reagent (Invitrogen, Cat. No. 15596018) in combination with the RNeasy Plus Mini Kit and RNase-free DNase set (Qiagen, Cat. No. 74104), following the manufacturer’s instructions with some modifications. Briefly, 200 mg of each SAT (*n* = 10) and VAT (*n* = 10) sample from each individual cow was immersed in a 2-mL RNase-free O-ring tube containing 1 mL of Trizol and 10 3-mm high-impact zirconium beads (Benchmark Scientific Inc., Cat. No. D1032-30). Tissue was homogenized using a beadblaster (Benchmark Scientific Inc., Sayreville, NJ, USA) for 10 min. After homogenization, the sample was centrifuged at 12,000 × *g* for 10 min at 4°C, and a pink middle layer fluid was collected into a new 2-mL RNase-free microtube. Then, 200 µL of phenol:chloroform (Invitrogen, Cat. No. AM9732) at 4°C was added to the sample to isolate the RNA from the organic phase. After centrifugation at 12,000 × *g* for 15 min at 4°C, the clear upper phase was transferred into a new 2-mL RNase-free microtube containing 700 µL of 70% molecular biology grade ethanol (Fisher Bioreagents, Cat. No. BP2818100). Total RNA was purified using the RNeasy Plus Mini Kit and eluted in 30 µL of RNase-free water. RNA integrity was assessed using the RNA Nano 6000 Assay Kit of the Bioanalyzer 2100 system (Agilent Technologies, Santa Clara, CA, USA). First strand cDNA was synthesized using random hexamer primer and M-MuLV Reverse Transcriptase (RNase H-). Second strand cDNA synthesis was subsequently performed using DNA Polymerase I and RNase H. PCR was performed with Phusion High-Fidelity DNA polymerase, Universal PCR primers, and Index (X) Primer. PCR products were purified (AMPure XP system) and library quality was assessed using the Agilent Bioanalyzer 2100 system. The clustering of the index-coded samples was performed on a cBot Cluster Generation System using TruSeq PE Cluster Kit v3-cBot-HS (Illumina, Inc., San Diego, CA, USA) according to the manufacturer’s instructions. After cluster generation, the library preparations were sequenced on an Illumina Novaseq 6000 S4 platform to generate 30 million stranded paired-end reads (2 × 150 nt) per sample. We obtained an average of 69.4 ± 9.1 (mean ± SD) million clean reads per sample.

#### Bioinformatics pipeline

Reads were trimmed for quality and adapters with TrimGalore 0.4.3 and mapped to bovine genome ARS-UCD1.2 with STAR-2.7.2a (28) in the two-pass mode, quantMode GeneCounts including the following specifications: –outSAMstrandField intronMotif –outFilterIntronMotifs RemoveNoncanonicalUnannotated –alignEndsType Local –chimOutType WithinBAM –twopassMode Basic –twopass1readsN -1. Annotation was performed using Ensembl v106. Normalization of expression values was performed using gene length corrected trimmed mean of M-values (GeTMM) (29). Differentially expressed genes (DEGs) were identified by one-way ANOVA with a Huber M-estimation to control for outliers. Significance between samples was based on a Benjamini–Hochberg corrected false discovery rate (FDR) *p*-value < 0.05. DEGs were graphically presented by principal component analysis (PCA) using JMP Pro 14 and by volcano plots using R package EnhancedVolcano version 1.10.0. Depot and SCK exclusively expressed genes were defined by GeTMM values greater than 0 for one group, while GeTMM values for its comparison group were equal to 0.

#### Functional genomics

Functional genomic analysis was performed using weighted gene co-expression network analysis (WGCNA; version 1.66 package in R) to construct gene co-expression networks (30, 31). WGCNA was performed by inputting the whole transcriptome from SAT and VAT datasets separately among experimental groups (SCK and NK). Only genes expressed in at least 50% of samples in each dataset were included in the analysis. The GeTMM values for each gene were log_2_ transformed to normalize the data. A pairwise correlation matrix was constructed between all pairs of genes across the samples, and a matrix of weighted adjacency was generated by raising co-expression to a power of 9, as determined for our sample set (30, 32). A topological overlap matrix (TOM) was then assembled and used as input for hierarchical clustering analysis. A dynamic tree cutting algorithm was used to identify gene clusters or modules (i.e., genes with high topological overlap) in an unsupervised fashion. Gene modules were visualized by a heatmap plot (TOMplot) of the gene network topological overlap. Module relationships were summarized by a hierarchical clustering dendrogram and TOMplot of module eigengenes (MEs). Associations between gene modules and traits of interest were tested by correlating MEs to trait score. Traits of interest used for WGCNA included the following: NK SAT, SCK SAT, NK VAT, and SCK VAT. Module–trait correlations were visualized using a heatmap plot and only modules with trait relationship significance (*R* ^2^) higher than 0.7 and *p*-value ≤ 0.05 were considered for further analysis. Module memberships [MM; correlation between each gene expression profile (GeTMM) and the ME of a given module as an indicator of the intramodular connectivity] and gene significance [GS; correlation between the gene expression profile (GeTMM) and the trait score as a measure of biological relevance] were calculated (30). Genes (network nodes) having MM ≥ 0.90 were identified as intramodular hub genes (33).

Using DAVID Bioinformatics Resources (version 6.8) (34), gene ontology (GO) analysis was performed using the entire gene list derived from each ME identified as described above (turquoise *p*-value *=* 0.02, brown *p*-value *=* 0.04, green *p*-value *=* 0.05, and blue *p*-value *=* 0.03) to functionally annotate their biological processes (BPs), while Ingenuity Pathway Analysis (IPA) identified activated and inhibited canonical pathways and upstream regulators (**Figure 1B**). Overrepresented BP analysis was performed on fold enrichment values with *p*-value < 0.01 (represented in bold in **Supplementary Table 4** **).** Fold enrichment was determined by comparing the background frequency of total genes annotated to a certain BP in the specified species genome to the sample frequency of genes under such BP (35). According to Menarim, El-Sheikh Ali (36), this approach takes advantage of the superior annotation of BPs from DAVID and the better pathway annotation of IPA. To predict upstream regulators relevant for each set of DEGs, we performed an analysis using the IPA software (37). The analysis provided a *p*-value of overlap, activation *Z*-scores, and the downstream targets for each predicted upstream regulator. *Z*-scores were used to predict the activation state (activation or inhibition) of each upstream regulator/signaling pathway. Predicted upstream regulators were considered significant if they had *p*-value ≤ 0.05 and activation *Z*-score >2 (activated) or >2 (activated). Subsequently, we investigated the overlap between the predicted upstream regulators for each set and the DEGs from the same set to identify potential regulators among those DEGs. Genes in common between the two analyses with *Z*-scores generated by IPA matching the direction of fold change (generated by DESeq2) were defined as potential regulators. To investigate the interaction and relationships between potential upstream regulators, all known protein–protein interactions were referenced and matched using STRING (version 10.5) (38). IPA was also used to determine activated and inactivated signaling pathways, considering significance at activation *Z*-score >2 (activated) or <−2 (suppressed).

### Immunohistochemistry

Aliquots from the SAT and VAT samples from each cow used for RNA sequencing analysis were formalin-fixed, paraffin-embedded, and sectioned at 4 µm and processed at the School of Veterinary Medicine and Biomedical Sciences Histology Lab at Texas A&M University (**Figure 1C**). Briefly, slides with two histological sections of each depot per animal were deparaffinized in xylene and rehydrated through a graded alcohol series. Antigen retrieval was performed in a pressure cooker (Decloaking Chamber, Biocare Medical, Pacheco, CA) using a pH 6.2 buffer (Diva, Biocare Medical). The procedure was run on an autostainer (intelliPATH FLX, Biocare Medical, Pacheco, CA). Target antibodies for the immunohistochemistry analyses were selected based on the identification of key genes in activated and inactivated canonical pathways, upstream regulators between SCK and NK in VAT and SAT samples, and markers of immune cell activation and infiltration in the AT. Selected primary antibodies were as follows: CD20 (Invitrogen, Cat. No. PA5-16701), CD3 (Agilent, Cat. No. A045229-2), IBA1 (Biocare, Cat. No. CP290A), CSFR1 (Invitrogen, Cat. No. PA5-14569), SPP1 (Bioss Antibodies, Cat. No. BS-0019R), FN1 (Invitrogen, Cat. No. FI24921), CD44 (Invitrogen, Cat. No. MA4400), TGFB1 (Invitrogen, Cat. No. MA1-21595), and BAFF-TNFSF13B (Invitrogen, Cat. No. PA1-41266). The primary antibodies were incubated for 30 min at room temperature followed by incubation with a polymer detection reagent (Mach 2 Rabbit HRP Polymer, Medical, Pacheco, CA) for 30 min. DAB chromogen was used to detect the sites of antibody–antigen interaction. Hematoxylin was used as the counterstain. A negative tissue control was run by substituting a negative isotype control for the primary antibody (**Supplementary Figure 1**). Finally, the slides were then dehydrated through graded alcohols and cleared with xylene.

Using the Pannoramic Scan II by 3DHistec, glass slides were scanned at 20× magnification, and the subsequent digital images were analyzed using Visiopharm Software (Version 2023.01 Hoersholm, Denmark) (39–41). First, the region of interest (ROI) was manually defined and measured (mm^2^). For the selected targets, a custom artificial intelligence-based Visiopharm analysis protocol package was designed to detect the total number of cells with positive labeling (positive cells) within the ROI. Immunohistochemistry data are presented as normalized to tissue area, in which the number of positive cells for determined antibody was divided by tissue area (mm^2^). Statistical analysis of IHC data was performed using the MIXED procedure of SAS 9.4 (SAS Institute Cary NC, USA), with a model containing the effects of depot, experimental group (NK or SCK), and their interaction as fixed effects. Statistical significance was declared at *p*-value ≤ 0.05 and tendencies were declared at *p*-value ≤ 0.10.

## Results

### Blood biomarkers

As expected, analytical chemistry revealed that cows with SCK had greater serum BHB (*p*-value < 0.01) concentrations compared to NK, confirming higher blood BHB concentrations detected by the handheld ketometer during animal selection. While serum NEFA concentrations did not differ between groups (*p*-value = 0.25), their concentrations in both SCK and NK indicated increased lipid mobilization when compared with reference values (**Table 2**). Serum concentrations of phosphorus (*p*-value = 0.03) were increased in SCK *vs.* NK, while serum concentrations of sodium, glucose, albumin, cholesterol, urea nitrogen, calcium, and potassium were similar between NK and SCK *(p*-value > 0.05).

**Table 2:** Metabolic profile of postpartum dairy cows assigned to subclinical ketosis (SCK) and non-ketotic (NK) experimental groups.

Metabolite	SCK	NK	SEM^1^	*p*-value
Glucose, mg/dL	41.8	43.6	3.37	0.72
BHB, mmol/L	1.06	0.68	0.05	<0.01
NEFA, mEq/dL	1.17	0.90	0.07	0.25
Albumin, mg/dL	3.22	3.36	0.11	0.39
Cholesterol, mg/dL	83.4	96.4	8.17	0.29
Blood urea nitrogen, mg/dL	11.8	11.2	0.50	0.43
Calcium, mg/dL	9.26	9.20	0.06	0.50
Sodium, mEq/dL	141.6	139.4	0.81	0.09
Potassium, mEq/dL	4.44	4.48	0.15	0.85
Phosphorus, mg/dL	5.50	4.48	0.23	0.03
Chlorine, mEq/dL	100.8	102.0	0.47	0.14

^1^ Largest standard error of the mean.

### Transcriptional profiles in VAT and SAT of dairy cows with and without subclinical ketosis

#### Differential gene expression

**Figure 2:**
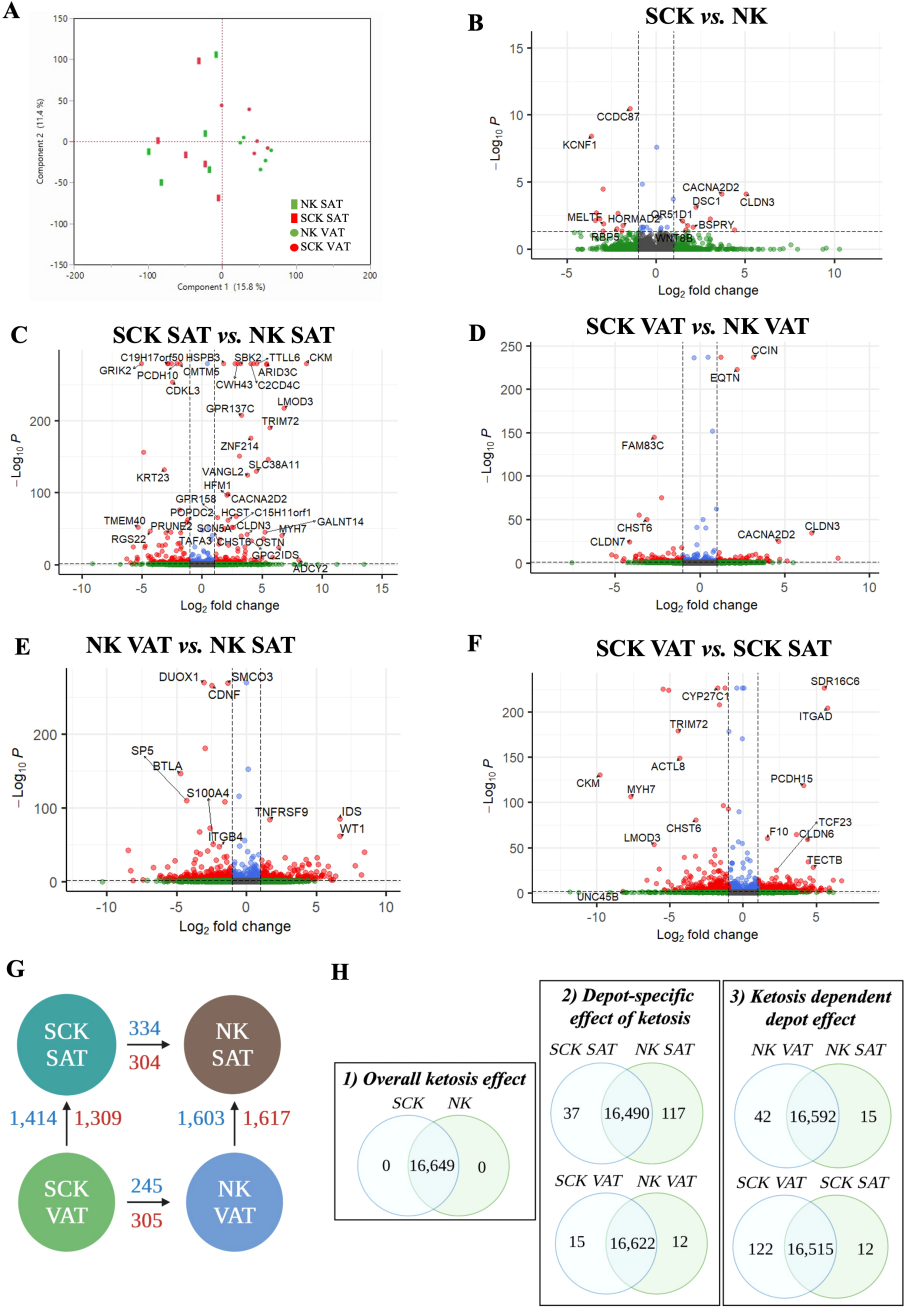
Gene expression contrasts. **(A)** Principal component analysis from differently expressed genes (DEGs) in NK SAT (green rectangle), SCK SAT (red rectangle), NK VAT (green circle), and SCK VAT (red circle). Each shape in the graph represents an individual sample. Volcano plots showing downregulated (red left quadrant) and upregulated (red right quadrant) DEGs in **(B)** SCK *vs*. NK, **(C)** SCK SAT *vs*. NK SAT, **(D)** SCK VAT *vs*. NK VAT, **(E)** NK VAT *vs*. NK SAT, and **(F)** SCK VAT *vs*. SCK SAT. **(G)** Schematic of differences in gene expression considering the studied contrasts, depicting the number of DEGs between SCK and NK within the same depot (SAT or VAT, horizontal comparisons), and between depots within the same condition (SCK or NK, vertical comparisons). The number of upregulated genes is represented in blue and downregulated genes are shown in red. Arrows indicate the sense of the comparison, e.g., SCK VAT showed 245 upregulated and 305 downregulated DEGs compared to NK VAT. DEG were identified by one-way ANOVA with a Huber M-estimation to control for outliers. Significance between samples was based on a Benjamini–Hochberg corrected false discovery rate (FDR) *p*-value < 0.05. The entire list of DEGs can be found in **Supplementary Table 1**. **(H)** Venn diagrams illustrating the intersection and uniqueness.

AT transcriptome analysis was performed to identify (1) the overall effects of ketosis independent of depot (SCK *vs.* NK), (2) the depot-specific effects of ketosis (SCK SAT *vs.* NK SAT and SCK VAT *vs.* NK VAT), and (3) the ketosis-dependent depot effects (SCK VAT *vs.* SCK SAT and NK VAT *vs.* NK SAT). The full list of DEGs for each contrast is reported in **Supplementary Table 1**. PCA of DEGs revealed only depot-related differences (**Figure 2A**). Volcano plots showed upregulated and downregulated DEGs for each contrast (**Figures 2B–F**), while the number of DEGs in each contrast is reported in **Figures 2G, H**. The most remarkable number of DEGs was observed when comparing depots (VAT or SAT) in NK or SCK animals (**Figure 2G**). We detected only 35 DEGs between SCK and NK across both depots (**Figure 2B**). The number of DEGs was increased 15-fold when contrasting SCK and NK within SAT or VAT (**Figures 2C, D, G**), demonstrating the depot specificity of SCK effects. However, there was great transcriptome overlap (>16,000 genes) between SCK SAT *vs.* NK SAT and SCK VAT *vs.* NK VAT (**Figure 2H**). A similar pattern of DEGs number was observed when contrasting VAT and SAT in SCK or NK cows separately.

**Figure 3:**
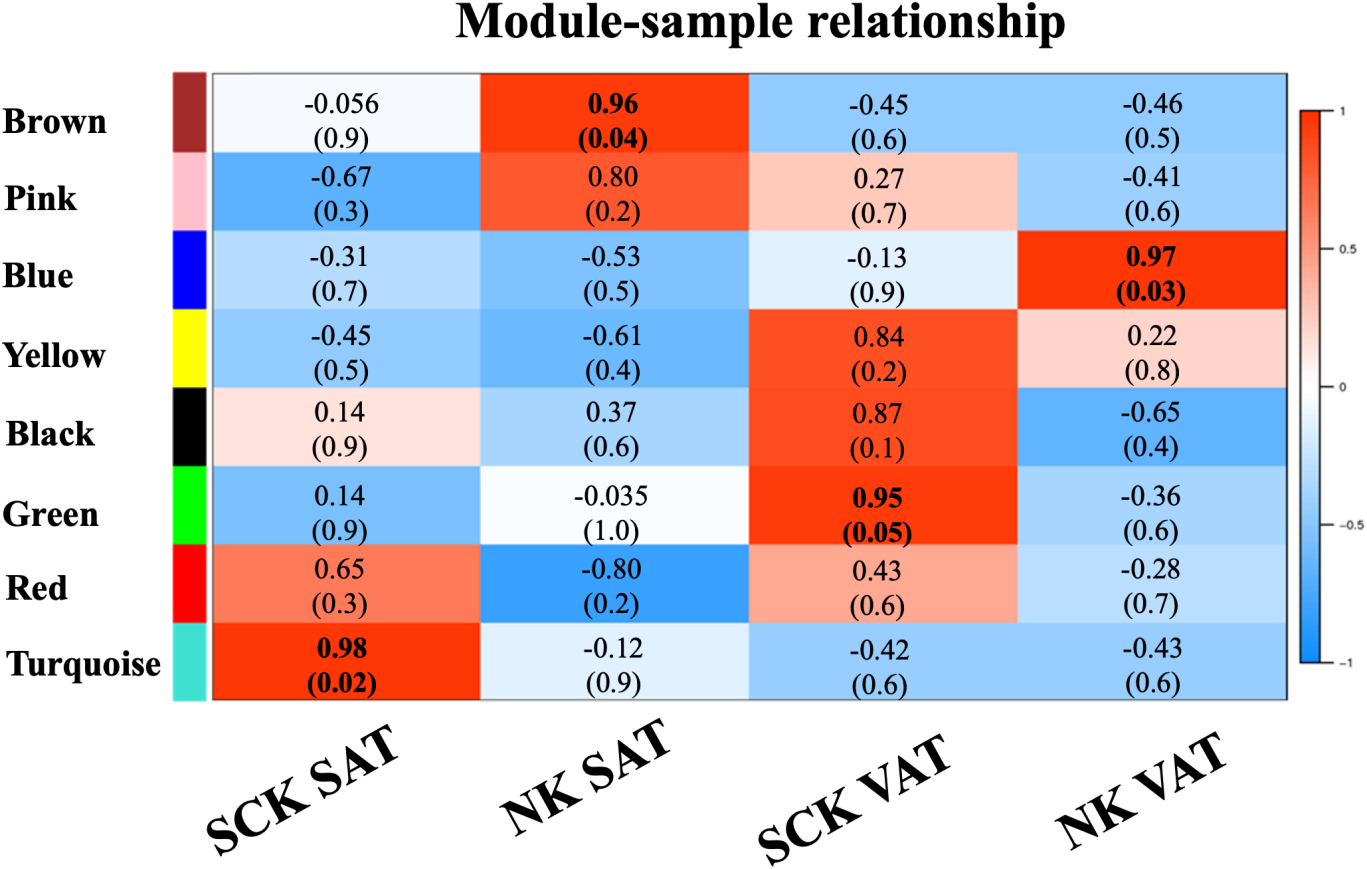
Weighted gene co-expression network analysis (WGCNA): Module–sample relationships. WGCNA of 16,649 genes in AT identified eight module eigengenes (ME), four of which exhibited positive association (*R*² ≥ 0.7, top; *p*-value ≤ 0.05, bottom between parenthesis) with the assigned traits (SCK SAT, NK SAT, SCK VAT, and NK VAT). The turquoise ME was associated with SCK SAT, the brown ME was associated with NK SAT, the green ME was associated with SCK VAT, and the blue ME was associated with NK VAT.

For each contrast, we also identified exclusively expressed genes in a depot- and disease-specific manner (**Supplementary Table 2**). While overall ketosis-specific genes across both depots were absent (**Figure 2H-1**), we identified ketosis-specific genes in SAT and VAT when analyzed separately (**Figure 2H-2**). Examples of exclusively expressed protein coding genes in SCK SAT included regulators of cell migration, such as *RGS11* and *COLEC10*, while *IF1DA6*, the chemotactic *S100A7*, and the insulin-sensitizing adipocyte-secreted protein *C1QTNF12* were uniquely expressed by NK SAT (**Supplementary Table 2**). In ketosis, the number of exclusively expressed genes was increased by 10-fold in VAT compared to SAT, and by nearly 3-fold in non-ketotic conditions (**Figure 2H-3**). Based on average GeTMM, the top protein coding genes uniquely expressed by ketotic VAT *vs.* SAT included markers of adipogenic inhibition [*DLK1* (Pref-1)] and mesothelial cells (*WT1* and *MSLN*), while markers of proliferation (*FOXL1* and *TNMD*) and angiogenesis (*ANGPTL5I*) were uniquely expressed by non-ketotic SAT (**Supplementary Table 2**).

#### Co-expression network analysis, modular gene ontology enrichment, and overrepresented biological processes

WGCNA provided further information regarding the patterns of gene co-expression and the identification of genes with the highest intramodular connectivity (hub genes, MM ≥ 0.90) in our dataset (denoted by bold font in **Supplementary Table 3**). Co-expression analysis of a total of 16,649 genes on SAT and VAT samples identified eight MEs or clusters (**Figure 3**), of which four were positively associated (*R*² ≥ 0.7 and *p*-value ≤ 0.05) with the assigned traits (SCK SAT, NK SAT, SCK VAT, and NK VAT). There were significant positive associations of the turquoise module with SCK SAT, the brown module with NK SAT, the green module with SCK VAT, and the blue module with NK VAT.

**Figure 4:**
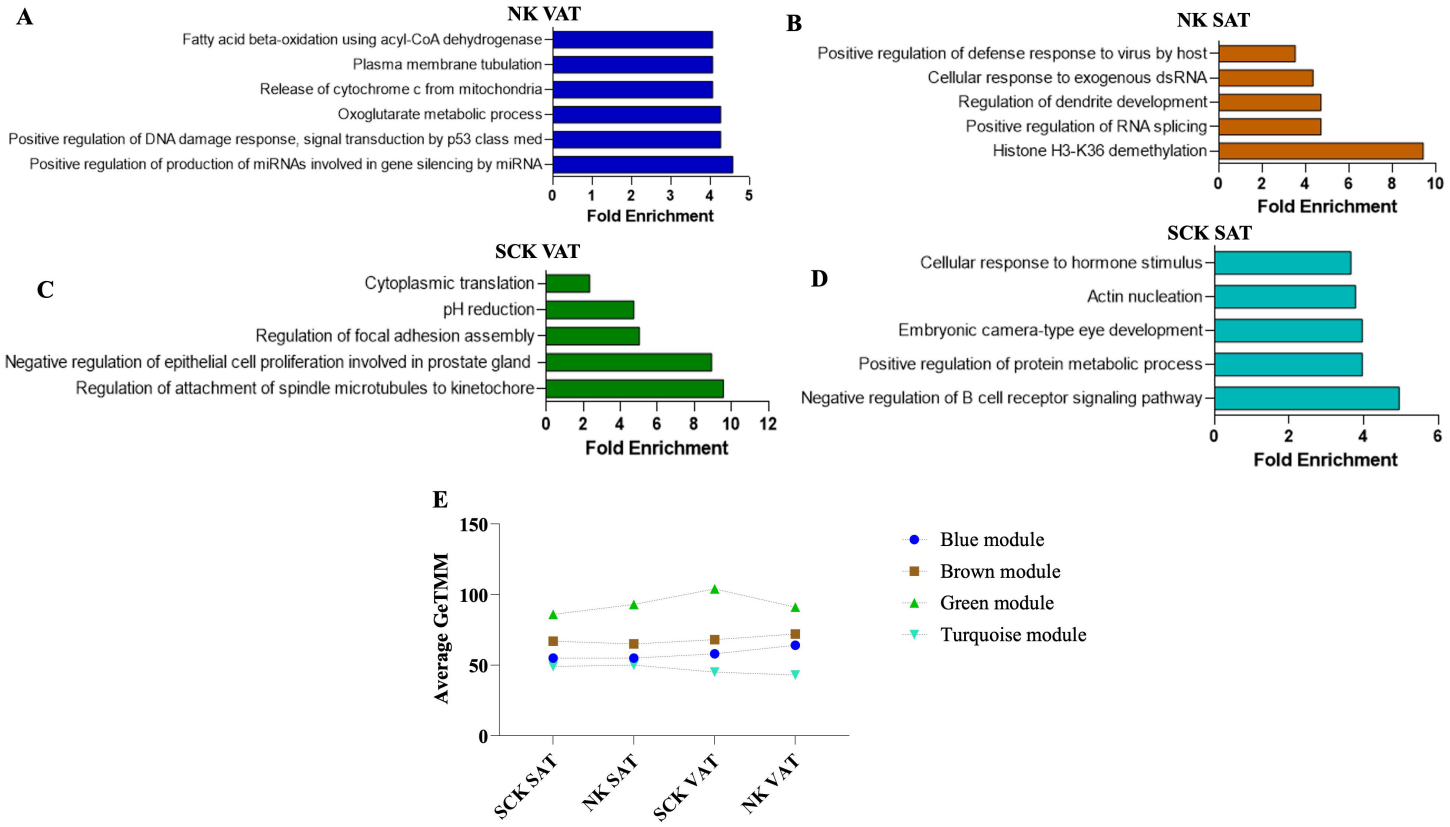
Expression profile, gene ontology enrichment, and overrepresented biological processes within dominant modules. The blue module was positively associated with NK VAT, the brown was positively associated with NK SAT, the green was positively associated with SCK VAT, and the turquoise was positively associated with SCK SAT. Top significant overrepresented Biological Processes (BP) in the **(A)** blue, **(B)** brown, **(C)** green, and **(D)** turquoise modules and their corresponding fold enrichment. **(E)** Mean expression profiles of significant modules considering genes with the highest intramodular connectivity (hub genes, R^2^ > 0.90) of each module. There was an increased mean expression of the homeostatic/pro-resolving blue and brown modules in NK samples **(A, B)**, while the pro-inflammatory green and turquoise modules were positively associated with SCK samples **(C, D)**. The complete list of significant BPs and their respective gene list are presented in **Supplementary Table 4**.

The analysis of overrepresented BPs for each significant module uniquely associated to SCK SAT, NK SAT, SCK VAT, and NK VAT provided additional functional analysis of gene clusters (**Figures 4A–D**; **Supplementary Table 4**). The top overrepresented BPs associated with NK AT are highlighted in **Figures 4A,B** and indicate depot-specific effective homeorhetic mechanisms by which AT remodels in response to increased lipid mobilization during postpartum. In NK VAT, overrepresented BPs and genes within each BP in the blue module suggest catabolic mechanisms via lipolysis and corresponding metabolic adaptations, including insulin signaling regulation, and cell and nucleic acid synthesis and repair (**Figure 4A** **;** **Supplementary Table 4**). Additional top BPs in the blue module associated with NK VAT included response to oxidative stress, regulation of canonical NF-κB signaling, and other BPs related to lipid metabolism, e.g., fatty acid beta-oxidation, acyl-CoA metabolic process, and fatty acid metabolic process (**Supplementary Table 4**), thus indicating an intense metabolic and pro-inflammatory remodeling within VAT in response to lipid mobilization, even in the absence of ketosis. In NK SAT, gene profiles from overrepresented BPs in the brown module reflect epigenomic regulations associated with homeostatic IL-12 signaling (evidenced by the gene profile of *Positive Regulation of Defense Response to Virus by Host* BP) and mitochondrial metabolism of fatty acids during postpartum adaptation (**Figure 4B** **;** **Supplementary Table 4**). Further top BPs in the brown module-associated NK SAT highlighted the involvement of distinct organelles (e.g., mitochondria, ER, and Golgi) in response to increased fatty acid metabolic processes in postpartum dairy cows in NEB. These differences between the transcriptional profile of VAT and SAT depict their distinct functional roles in postpartum metabolic adaptations.

Green and turquoise modules were associated with SCK VAT and SCK SAT, respectively (**Figures 4C,D**). The top five overrepresented BPs in the green module associated with SCK VAT highlight changes on focal adhesion, cell proliferation, and protein translation (**Figure 4C**) driven by the expression of many genes related to cell cycle (e.g., *CDC42, APC*, and *STK11*), triacylglycerol synthesis and storage (*LPIN1* and *LPIN3*), cytoskeleton assembly (e.g., *ACTR3, ACTR2*, and *ACTG1*), and ATPases involved in organelle acidification (e.g., *ATP6V1E1*, *ATP6V1H*, and *ATP6V0D1*) (**Supplementary Table 4**). Notably, the regulation of inflammatory responses, cell migrations, and positive regulation of interleukin-6 production were among the top BPs associated with SCK VAT mediated by the expression of genes such as *CCL3, CD63, THBS1, TNF*, and *TLR9*. These findings highlight marked inflammatory responses and immune infiltration, specially mediated by endocytic processes (phagocytic and pinocytic), phagolysosome maturation events, and apoptosis (**Supplementary Table 4**).

In SCK SAT, the top BP in turquoise module highlights *Negative Regulation of B-Cell Receptor Signaling* mediated by the expression of genes, such as *CD22* and *GPS2*, the latter being a key regulator of inflammation, lipid metabolism, and mitochondrion homeostasis, and a coactivator for the recruitment of PPARG in adipocytes (**Figure 4D**). These immune-metabolic changes in SCK SAT were also evidenced by the *Positive Regulation of Protein Metabolic Processes* GO term, which involved genes regulating the production of lipoproteins (i.e., *NR1H2*), adipocyte differentiation (i.e., *MTPN* and *UHRF1*), and the activation, proliferation, and migration of immune cells (i.e., *KLF2*) (**Figure 4D** **;** **Supplementary Table 4**). Interestingly, the *Embryonic Camera-Type Eye Development* GO term highlights the expression of *RARG* and *IFT140*, emphasizing the involvement of membrane–ECM interactions in SCK SAT (**Supplementary Table 4**). Furthermore, BPs of *Cellular Response to Hormone Stimulus* included *NCOA1* and *NCOA2*, both nuclear receptor co-activators with epigenomic regulatory functions, and *RAMP* (2 and 3), which have immunomodulatory functions related to endothelial cells. *RAMP* genes were also evidenced in other significant GO terms in SCK SAT including vasculogenesis, angiogenesis, and protein localization to plasma membrane underscoring the evident effect of SCK on SAT vascular function (**Supplementary Table 4**).

To assess how each significant module (cluster of co-expressed genes) changed across study groups, the mean expression profile (mean GeTMM values for all genes within a given module) of each module was plotted for each study group/trait (**Figure 4E**). The patterns of expression for the brown and blue modules associated with NK SAT and NK VAT (healthy state) showed overall overlapping patterns across groups with higher expression in VAT. The green module associated with SCK VAT exhibited the highest magnitude of expression across all groups. Noticeably, while it most highly expressed in SCK VAT, its lowest expression values were for SCK SAT, reflecting opposing effects of ketosis in the AT depots studied. Finally, the turquoise module, which associates with SCK SAT, exhibited the lowest expression profiles across groups and no pattern of expression relating to SCK or depot was identified (**Figure 4E**).

#### Pathway analysis, upstream regulators, and predicted network interactions

**Figure 5:**
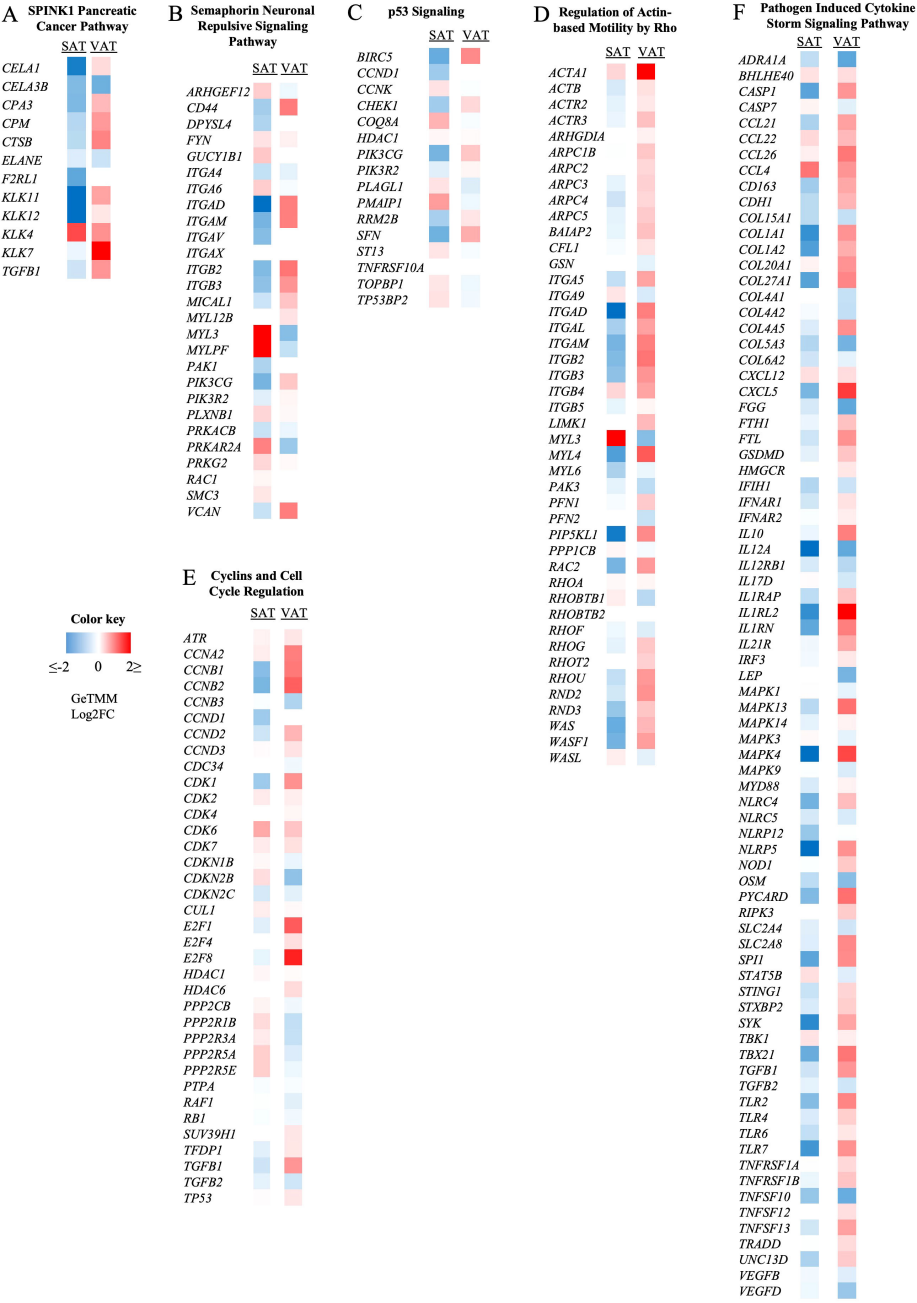
Effect of subclinical ketosis on the expression patterns of genes involved in different signaling pathways. Colors represent the fold change (GeTMM Log2FC) of gene expression in subclinical (SCK) compared to non-ketotic (NK) samples in subcutaneous (SAT, left column) and visceral (VAT, right column) AT. Red shows gene upregulation in SCK, while blue shows downregulation in SCK when in contrast with NK. SPINK1 pancreatic cancer **(A)**, semaphorin neuronal repulsive **(B)**, and p53 signaling **(C)** are the most activated pathways in SAT when comparing SCK to NK. Regulation of actin-based motility by rho **(D)**, cyclins and cell cycle regulation **(E)**, and pathogen-induced cytokine storm signaling pathway **(F)** are the most activated pathways in VAT when comparing SCK to NK. The full list of activated and inactivated pathways is available in **Supplementary Table 5**.

IPA revealed activated and inactivated pathways in most of the studied comparisons (**Table 3**, **Supplementary Table 5**), with the exception of SCK *vs.* NK overall comparison, in which the number of DEGs was too low to yield conclusive results. Using the top three most activated pathways comparing SCK *vs.* NK in SAT or VAT, we created heatmaps depicting the expression patterns of genes involved in each pathway, which provided further insights into mechanisms underlying the depot-specific effects of SCK in AT (**Figure 5**).

**Table 3:** Top five most activated and inactivated pathways (Z-score > 2.0 or <−2.0) identified by IPA from DEGs in the studied contrasts.

Ingenuity canonical pathways	Z-score	−Log (p-value)
SCK SAT vs. NK SAT		
Activated		
SPINK1 Pancreatic Cancer Pathway	2.887	1.97
Semaphorin Neuronal Repulsive Signaling Pathway	2.449	2.98
p53 Signaling	2.333	1.61
LXR/RXR Activation	2.309	0.53
SNARE Signaling Pathway	2.132	1.97
Inactivated		
Pathogen-Induced Cytokine Storm Signaling Pathway	−3.709	2.61
Dendritic Cell Maturation	−3.402	0.00
GP6 Signaling Pathway	−3.272	4.63
Role of Osteoclasts in Rheumatoid Arthritis Signaling Pathway	−3.130	4.07
TREM1 Signaling	−3.051	1.50
SCK VAT vs. NK VAT		
Activated		
Regulation of Actin-based Motility by Rho	3.536	5.13
Cyclins and Cell Cycle Regulation	3.528	5.59
Pathogen-Induced Cytokine Storm Signaling Pathway	3.145	0.83
Actin Nucleation by ARP-WASP Complex	3.138	6.80
Epithelial Adherens Junction Signaling	2.940	4.78
Inactivated		
Tryptophan Degradation III (Eukaryotic)	−3.464	3.62
Valine Degradation I	−3.207	7.00
Glutaryl-CoA Degradation	−3.000	3.31
Xenobiotic Metabolism AHR Signaling Pathway	−2.985	0.70
Coronavirus Pathogenesis Pathway	−2.967	5.75
SCK VAT vs. SCK SAT		
Activated		
Oxidative Phosphorylation	4.990	6.20
Superpathway of Cholesterol Biosynthesis	3.771	3.34
EIF2 Signaling	3.732	9.32
Immunogenic Cell Death Signaling Pathway	3.528	0.39
Th1 Pathway	3.202	2.48
Inactivated		
Senescence Pathway	−2.928	9.23
TNFR2 Signaling	−2.840	1.41
Protein Kinase A Signaling	−2.811	11.20
CD27 Signaling in Lymphocytes	−2.600	2.96
Calcium Signaling	−2.517	4.12
NK VAT vs. NK SAT		
Activated		
Superpathway of Cholesterol Biosynthesis	4.025	4.73
Valine Degradation I	3.873	5.97
TCA Cycle II (Eukaryotic)	3.873	2.77
Isoleucine Degradation I	3.464	5.79
Fatty Acid β-oxidation I	3.441	4.06
Inactivated		
Pathogen-Induced Cytokine Storm Signaling Pathway	−3.846	0.42
Signaling by Rho Family GTPases	−3.702	120
IL-15 Production	−3.500	5.23
Dendritic Cell Maturation	−3.204	0.00
Role of PKR in Interferon Induction and Antiviral Response	−3.151	3.80
VAT vs. SAT		
Activated		
Superpathway of Cholesterol Biosynthesis	3.638	7.39
Fatty Acid β-oxidation I	3.207	3.69
Isoleucine Degradation I	3.162	5.57
Superpathway of Geranylgeranyldiphosphate Biosynthesis I (via Mevalonate)	3.000	3.76
Valine Degradation I	3.000	3.06
Inactivated		
Synaptic Long-Term Depression	−2.967	1.58
TNFR1 Signaling	−2.530	0.49
NER (Nucleotide Excision Repair, Enhanced Pathway)	−2.500	0.00
Semaphorin Neuronal Repulsive Signaling Pathway	−2.469	5.72
TNFR2 Signaling	−2.449	0.36

In SAT, SCK activated pathways involved the regulation of immune and inflammatory responses, and ECM remodeling (**Supplementary Table 5**). The top identified pathways included the *SPINK1 Pancreatic Cancer Pathway* (**Figure 5A**), *Semaphorin Neuronal Repulsive Signaling Pathway* (**Figure 5B**), p53 Signaling (**Figure 5C**), and *LXR/RXR activation*. Collective assessment of the gene profiles identified in the referred pathways reflects the breakdown of AT and lipid metabolism. Interestingly, SCK *vs.* NK comparison in SAT samples reveals that during ketosis, most of the genes within the *SPINK1 Pancreatic Cancer Pathway* relate to AT breakdown and are comparatively upregulated in VAT and downregulated in SAT (**Figure 5A**), emphasizing depot-specific events of lipid mobilization during SCK. Accordingly, the gene pattern of the *Semaphorin Neuronal Repulsive Signaling Pathway* activated in SCK SAT (**Figure 5B**) suggests increased cell–cell and cell–matrix interactions (*ARHGF12, DPYSL4 VCAN*, and several integrins) (42) with increased infiltration of pro-resolving macrophages, as evidenced by markers of immune cell transmigration (*GUCY1b1* and *MYL3*), anti-inflammatory cytokine production (*PRKARA2* and genes depicted in the LXR/RXR pathway), and ECM remodeling (*MYL3* and *MYLPF*). The activation profile of genes within the *p53 Signaling Pathway* (**Figure 5C**) in SCK SAT reflects a halt in the cell cycle with suppressive effects on apoptosis (*BIRC5* and *COQ8A*), cell proliferation (*CCND1, CCNK*, and *SFN*), and AT inflammation (*PIK3CG* and *PIK3R2*), which might be associated with a response to the increased lipid metabolization in VAT (**Supplementary Table 5**, **Table 3**) (43, 44).

In VAT (**Supplementary Table 5**, **Table 3**), *Regulation of Actin-based Motility by Rho* was the most activated pathway in SCK (**Figure 5D**), followed by the *Cyclins and Cell Cycle Regulation Pathway* (**Figure 5E**) and the *Pathogen Induced Cytokine Storm Signaling Pathway* (**Figure 5F**). The gene patterns of the *Regulation of Actin-based Motility by Rho Pathway* revealed a marked upregulation of actin and actin-related proteins, myosin, and integrin genes, crucial for the effectiveness of immune cellular processes, such as cell motility, endocytosis, and chemotaxis (45, 46). The activation of the *Cyclins and Cell Cycle Regulation Pathway* highlights a potential stimulatory effect of ketosis on VAT cell proliferation, likely immune cells driving inflammatory responses, as implied by the combined upregulation of *TGFB1* (**Figure 5E**) and the *Pathogen-Induced Cytokine Storm Signaling Pathway* (**Figure 5F**). For the latter, the gene profile revealed a marked upregulation on the expression of chemokines (e.g., *CCL21, CLL22, CCL26*, and *CXCL5*), immune cell markers, including monocytes, macrophages, lymphocytes, dendritic cells (e.g., *CD163, IL10, TGFB1, IL12A*, and *IRF3*), and pro-inflammatory markers, such as toll-like receptors, tumor necrosis factor receptors, and inflammasome markers (NLR gene family) in VAT. These findings indicate a ketosis-driven inflammatory response in VAT of postpartum dairy cows, which is further supported by the activation of additional inflammatory pathways, such as IL-8 signaling, phagosome formation, and dendritic cell maturation (**Supplementary Table 5**). Of note, while *Regulation of Actin-based Motility by Rho* was the most activated pathway in SCK VAT and the *Pathogen-Induced Cytokine Storm Signaling Pathway* was the most inactivated pathway in SCK SAT (**Supplementary Table 5**), genes involved in each of these pathways were more highly expressed in VAT, once again underscoring the depot-specific effect of ketosis in AT, with special inflammatory responsiveness to VAT.

We also evaluated the ketosis-dependent (SCK VAT *vs.* SCK SAT and NK VAT *vs.* NK SAT) and ketosis-independent depot effects (VAT *vs.* SAT) (**Table 3**, **Supplementary Table 5**). In all comparisons, *Superpathway of Cholesterol Biosynthesis* was among the top two most activated pathways in VAT compared to SAT. Additionally, the top 10 most activated pathways in VAT included other energy/fatty acid-related oxidation and biosynthesis pathways. Of note, different from NK VAT *vs.* NK SAT and VAT *vs.* SAT comparisons, in ketotic conditions (SCK VAT *vs.* SCK SAT), the activation of inflammatory and immune response-related pathways among the top 10 pathways was evident in VAT, but not in SAT, and included the *Immunogenic Cell Death Signaling Pathway, Th1 Pathway*, and *Inflammasome Pathway*. These results confirm that ketosis triggers more pronounced immune and inflammatory responses in VAT than SAT.

**Figure 6:**
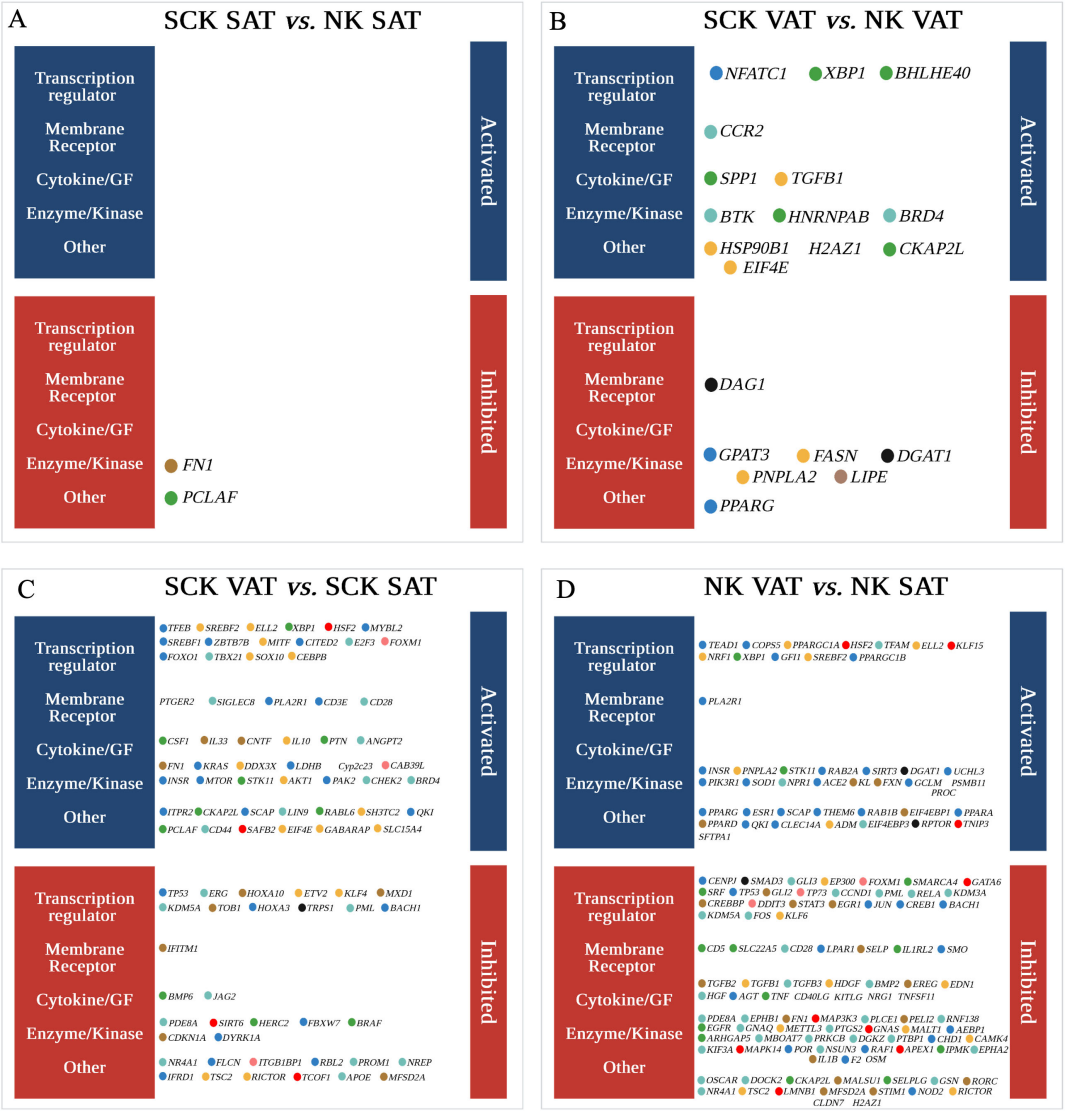
Potential upstream regulators of genes differentially expressed in **(A)** SCK SAT *vs.* NK SAT, **(B)** SCK VAT *vs.* NK VAT, **(C)** SCK VAT *vs.* SCK SAT, and **(D)** NK VAT *vs.* NK SAT. Upstream regulators were identified by analysis of the DEGs using Ingenuity Pathway Analysis (IPA). Colored circles are upstream regulators identified for each comparison with an activation or inhibition *Z*-score (>2.0 or <−2.0), respectively (**Supplementary Table 6**). The colors of the circles correspond to the colors of the module eigengene from WGCNA to which that specific gene is associated. Upstream regulators were grouped into five categories: transcription regulator, membrane receptor, cytokine/growth factor (GF), enzyme/kinase, and other.

**Figure 7:**
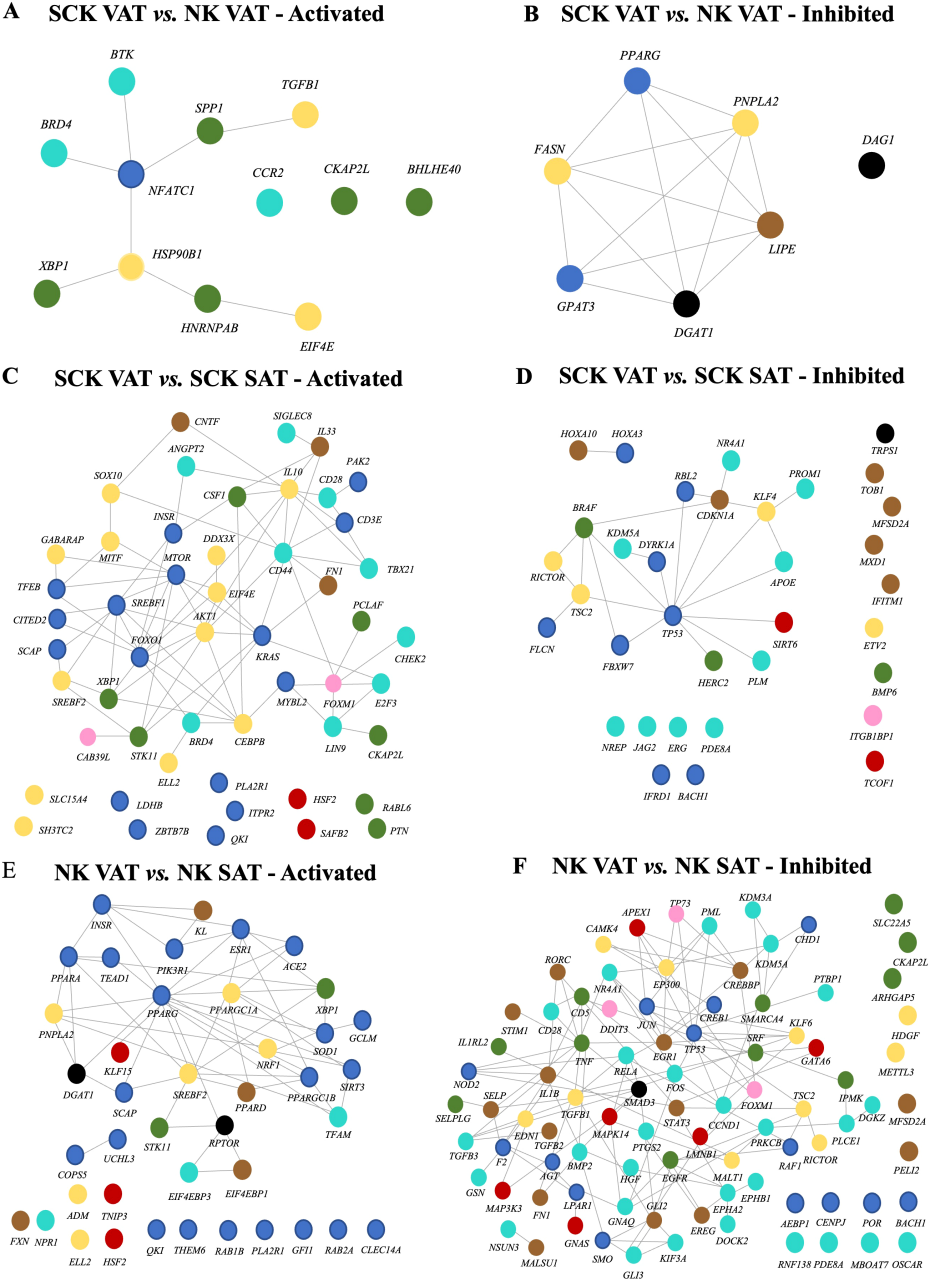
Interaction network among activated (left) and inhibited (right) upstream regulators based on the effects of ketosis within VAT **(A, B)** and ketosis-dependent **(C, D)** and -independent **(E, F)** effects on depot. Color coding of nodes relate to the corresponding module eigengene from WGCNA.

Analysis of the potential upstream regulators of genes differentially expressed between SCK and NK within depots, and between VAT and SAT in NK or SCK is shown in **Figure 6**. We identified only two inhibited (*p*-value <0.05 and a *Z*-score ≤−2) and zero activated (*p*-value <0.05 and a *Z*-score ≥2) upstream regulators in SCK SAT *vs.* NK SAT (**Figure 6A**). In SCK VAT *vs.* NK VAT, we revealed 7 inhibited and 13 activated upstream regulators (**Supplementary Table 6**, **Figure 6B**). Genes of the green module, including *SPP1, XBP1, CKAP2L, BHLHE40*, and *HNRNPAB*, were the most overrepresented (42%) among the activated upstream regulators in VAT of SCK *vs.* NK cows. Genes of the turquoise (*BTK, BRD4*, and *CCR2*) and yellow (*TGFB1, HSP90B1*, and *EIF4E*) modules followed with 25% each and finally that from the blue module (8%, *NFATC1*). In agreement with the IPA, activated upstream regulators in SCK VAT underpin the activation pro-inflammatory responses (*CCR2, SPP1*, and *TGFB1*). Taken together with the analysis of interaction networks activated in SCK VAT *vs.* NK VAT (**Figure 7A**), it further implies that *NFATC1*, the nuclear factor of activated T cells 1, is a key player in regulating VAT immune responses triggered by ketosis, which involves the regulation of B-cell development and maturation (*BTK*), cytokine activity by lymphoid and myeloid cells (*SPP1* and *TGFB1*), and protein folding and synthesis associated with inflammation and cellular stress (*HSP90B1* and downstream regulators). Among the inhibited upstream regulators in VAT of SCK *vs.* NK cows, blue (29%, *PPARG* and *GPAT3*), yellow (29%, *FASN* and *PNPLA2*), and black modules (29%, *DAG1* and *DGAT1*) were equally represented, followed by brown (14%, *LIPE*) (**Figure 6B**). Overall, the identification of these upstream regulators and their network interactions highlighted the inhibition of regulators of adipogenesis (*PPARG*), lipogenesis (*FASN, DGAT1*, and *GPAT3*), and lipolysis (*LIPE* and *PNPLA2;* **Figure 6B**), all interacting among each other without a central regulator (**Figure 7B**). Altogether, these findings imply that homeostatic mechanisms in response to ketosis may favor immune cell-mediated lipolysis in VAT while limiting AT expansion in both SAT and VAT.

While no upstream regulators were observed in the overall SCK *vs.* NK comparison, there was an increased number of upstream regulators comparing VAT *vs.* SAT in either ketotic or non-ketotic conditions (**Figures 6C, D**). In SCK cows, VAT *vs.* SAT comparison revealed 53 activated and 34 inhibited upstream regulators (**Figure 6C**), while in NK cows, the same comparison yielded 44 activated and almost 100 inhibited upstream regulators (**Supplementary Table 6**, **Figure 6D**).

In SCK VAT *vs.* SCK SAT, interaction networks revealed that among activated upstream regulators, genes of the blue module were the most overrepresented (31%, e.g., *mTOR, SREBF1*, and *CD3E*), followed by those of the yellow module (24%, e.g., *IL10, CEBPB*, and *SREBF2*) (**Figure 6C**). Accordingly, interaction network analysis of activated upstream regulators revealed predominant connections between genes from the blue and the yellow modules in SCK VAT vs. SCK SAT (**Figure 7C**). In contrast, among the inhibited upstream regulators, the turquoise module (26%, e.g., *APOE* and *ERG*) was the most overrepresented, followed by the blue module (24%, e.g., *TP53* and *IFRD1*) (**Figure 6C**). NK and SCK cows shared 18 upstream regulators in VAT *vs.* SAT, including the activation of *SREBF2*, a regulator of cholesterol biosynthesis and homeostasis, and *XBP1*, a modulator of cellular responses during endoplasmic reticulum stress (47) and B-cell differentiation (48), in addition to the inhibition of *TP53*, involved in suppressing cell division and inducing apoptosis (**Figures 6C, D**). Remarkably, interaction network analysis by STRING revealed *TP53* as a central upstream regulator inhibited in VAT *vs.* SAT of ketotic cows, in agreement with other IPA findings in VAT (**Figure 7D**). *TP53* was predicted to interact with most of the identified upstream regulators affecting cell differentiation, proliferation and apoptosis (e.g., *PROM1*), inflammatory response in macrophages, inhibition of NF-kappa-B signaling (e.g., *SIRT6* and *NR4A1*), and mTOR regulation (e.g., *RICTOR* and *FLCN*) (**Figure 6C**).

Finally, in NK VAT vs. NK SAT, among the activated upstream regulators, blue module was the most overrepresented, with more than 50% of genes (e.g., *PPARG, PPARA*, and *SOD1*), while among the inhibited upstream regulators, the turquoise module was the most overrepresented (32%, e.g., *CD28* and *FOS*) (**Figure 6D**). Interestingly, *CD28*, *CKAP2L*, *FN1*, and *FOXM1* were activated in VAT *vs.* SAT of ketotic animals but inactivated in non-ketotic conditions (**Figures 6C,D**). These genes are particularly important for T-cell activation and cellular processes underlying cell division, migration, and tissue repair involving ECM remodeling, and suggest potential VAT homeostatic mechanisms in response to ketosis. Furthermore, **Figure 7E** highlights *PPARG* and *PPARGC1A* as central activated upstream regulators in VAT *vs.* SAT of non-ketotic cows, interacting with lipid metabolism regulators (*PNPLA2, DGAT1*, and *SREBF2*) and mediators of insulin signaling (*INSR* and *PIK3R1*). Notably, there is an inhibition of key upstream regulators mediating pro-inflammatory responses, including *TNF, IL1B, RELA*, and *TGFB1* (**Figure 7F**). These results suggest key target genes and mechanisms underlying AT homeorhetic responses to postpartum NEB in the absence of ketosis in VAT *vs.* SAT. Overall, these results show disease- and depot-specific effects on tissue transcriptome that highlight the pro-immunogenic potential of VAT compared to SAT, especially in ketotic conditions.

### Adipose tissue expression of markers related to immune cell activation and infiltration

**Figure 8:**
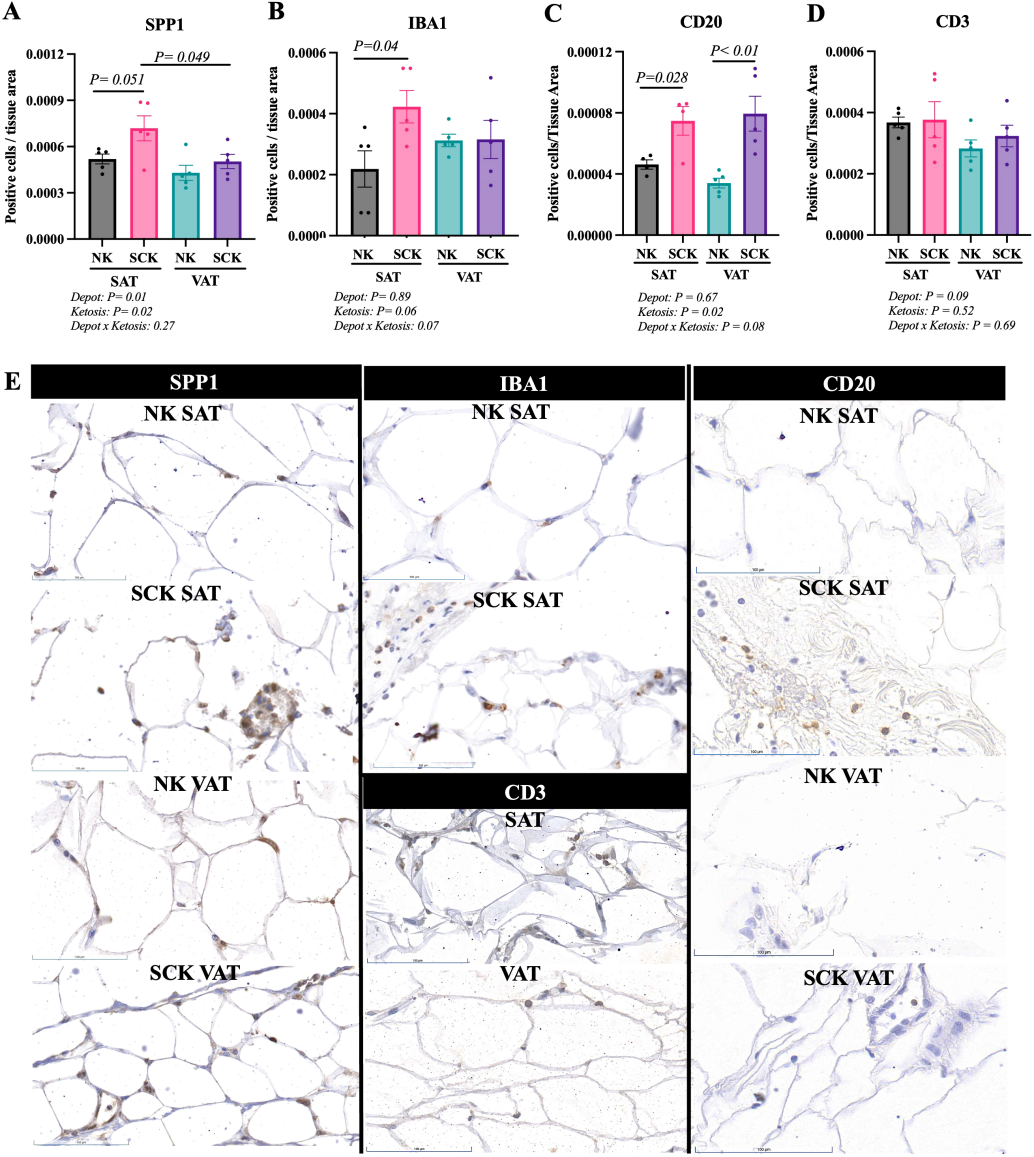
Immunohistochemistry of subcutaneous AT (SAT) and visceral AT (VAT) of postpartum dairy cows with subclinical ketosis (SCK) and non-ketotic (NK) animal. Scatterplots for SPP1 **(A)**, IBA1 **(B)**, CD20 **(C)**, CD3 **(D)**. Data are presented as number of positive cells normalized to tissue area (mean ± SEM). Each dot in the scatterplot represents the results for each individual dairy cow. **(E)** Representative sections of SAT and VAT of NK and SCK dairy cows for each marker analyzed. Positive and negative controls are shown in **Supplementary Figure 1**.

Target antibodies for the immunohistochemistry analyses were selected based on the identification of key genes in activated and inactivated canonical pathways, upstream regulators between SCK and NK within VAT and SAT samples, and markers of immune cell activation and infiltration in the AT. Patterns of expression for SPP1, IBA1, CD20, and CD3 and representative IHC images are presented in **Figure 8**. Antibodies used for FN1, CD44, TGFB1, and BAFF-TNFSF13B were not suitable for IHC under the tested conditions and did not yield any results. Osteopontin (SPP1) is a marker of activated macrophages, dendritic cells, and lymphocytes (49) and was identified as an activated upstream regulator in VAT of SCK *vs.* NK (**Figure 6B**). The abundance of SPP1-positive cells was increased on SAT *vs.* VAT, in SAT of SCK *vs.* NK, and in SCK SAT *vs.* SCK VAT (**Figures 8A, E**). The contrasting data between gene and protein expression suggest that SPP1 expression is regulated at distinct levels in SAT *vs.* VAT in postpartum cows. The ionized calcium binding adaptor molecule 1 (IBA1) is a protein found in macrophages and microglia that helps with membrane ruffling and phagocytosis, and its expression increases when these cells are activated (50). We observed an increase in the number of IBA1^+^ cells in SCK SAT compared to NK SAT, suggesting a higher number of activated macrophages on SAT of cows with SCK (**Figures 8B, E**). We also observed a direct effect of SCK on the number of CD20-expressing cells, a marker for B cells, in both SAT and VAT (**Figures 8C, E**), but no effects were observed on the abundance of CD3-positive cells, used as a marker of T cells (**Figures 8D, E**). Interestingly, there was a tendency of CD3-positive cells to be more abundant on SAT *vs.* VAT.

## Discussion

In our study, we defined the transcriptional profile and the expression of immune cell markers of VAT and SAT of postpartum dairy cows with SCK, one of the most prevalent and costly metabolic disorders in early lactation (17, 51). Our findings highlight a clear depot-specific effect of ketosis, which was nearly absent when depot was not considered. Our results illustrate that major changes resulting from SCK are particularly identified in VAT. The innate pro-inflammatory potential of VAT (22) in postpartum dairy cows in NEB is intensified by ketosis, while SAT demonstrated more homeostatic events in SCK. Changes in AT immune responses in ketotic conditions were accompanied by marked lipid catabolism in VAT and ECM remodeling, mostly observed in SAT. These key findings highlight differential homeostatic mechanisms in response to ketosis dependent on the anatomical location of AT and confirm findings from earlier research (21, 52, 53) and recent literature reviews (54, 55) that SAT is metabolically less responsive and more stable during the transition period, while VAT is more dynamic and may contribute more significantly to metabolic and inflammatory disturbances.

We observed serum NEFA concentrations greater than 0.9 mEq/dL in both SCK and NK cows, which are above critical cutoff points associated with increased risk for metabolic and inflammatory diseases (12). Although these results indicate that both groups had increased lipid mobilization, as expected in early lactation, BHB concentrations of NK cows were lower than the thresholds defined for SCK and indicate that not all postpartum animals with increased lipid mobilization will develop ketosis as it will depend on their ability to adapt to the NEB. The DEGs and the analysis of upstream regulators comparing VAT and SAT in the absence of ketosis suggest that VAT has a greater pro-adipogenic transcriptional potential compared to SAT as denoted by the expression of adipogenesis markers, such as Pref-1, and the activation of master regulators of adipogenesis and insulin signaling. Notably, ketosis shifts this profile by markedly inhibiting upstream regulators of adipogenesis and lipogenesis in VAT (e.g., *PPARG* and *FASN*) and by simultaneously activating markers of pro-inflammatory response (e.g., *CSF1* and *IL33*) and its resolution (e.g., *IL10* and *ANGPT2)*. As serum NEFA concentrations and other markers of systemic metabolism did not vary between NK and SCK cows, it suggests that the effects observed in AT are associated with hyperketonemia.

In our study, we used functional genomics (WGCNA combined with DAVID and IPA) to identify BPs and activated/inhibited signaling pathways and upstream regulators associated with ketosis and learned that it should be studied in a depot-specific manner. The depot-independent analysis of SCK *vs.* NK cows yielded a short list of DEGs, and no potential upstream regulators or activated and inactivated pathways were identified by IPA. This contrasted with SCK *vs.* NK analysis in a depot-dependent manner, which revealed a significantly higher number of DEGs and pathways. These results corroborate with the distinct cellular profile, and immunological and metabolic characteristics of SAT and VAT (22, 56), and show depot-specific responses to metabolic disorders, such as ketosis.

Using WGCNA and comparative transcriptome analyses, we identified a positive association between SCK VAT and the green module, in which *SPP1* was one of the genes with the highest intramodular connectivity (hub gene, *R*² > 0.90). SPP1, also known as osteopontin, is a multifunctional protein expressed in, e.g., activated immune cells (macrophages and T cells), osteoclasts, and endothelial cells, and its main function is to promote cell adhesion and to facilitate cell migration or survival (57). In tailhead SAT of dairy cows, *SPP1* increased approximately 10 days postpartum compared to prepartum (11) and was positively associated with AT macrophage infiltration, evidencing its chemotactic role (10). Notably, at the protein level, our IHC results demonstrate increased abundance of SPP1-positive cells in SAT of SCK *vs.* NK cows, but not in VAT, suggesting that increased expression may be an adaptive response to ketosis in SAT. In VAT, the identification of *SPP1* as an activated upstream gene regulator in SCK linked to *TGFB1* and *NFATC1* expression suggests significant AT remodeling and immune modulation in VAT, particularly in the context of macrophage, T-cell, and adipocyte interactions (10, 58). Accordingly, the green module also revealed an overrepresentation of the negative regulation of epithelial cell proliferation involving high *SERPINF1* expression (encoding the pigment epithelium-derived factor protein), which promotes lipolysis and has been linked to insulin resistance in human adipocytes (59, 60). Interestingly, other overrepresented BPs in the green module associated with SCK VAT also implied a potential detrimental effect on insulin signaling mediated by enriched genes, such as *APP* (amyloid-beta precursor protein) and *EGFR* (epidermal growth factor receptor). While *APP* mediates adipocyte mitochondrial dysfunction that leads to adipocyte hypertrophy and insulin resistance (61), *EGFR* has been linked to adipose insulin resistance led by the activation of AT macrophages in mice (62), thus highlighting the potential link between adipocyte dysfunction and immune cell activation in SCK cows, particularly in VAT.

The association of SCK SAT with the turquoise module highlights a link between ketosis and inflammatory response, adaptive immunity, and changes in vascular cells, but also with BPs that included the expression of many toll-like receptor genes (*TLR10, TLR7, TLR5, TLR3*, and *TLR2*), members of the tumor necrosis factor receptor superfamily (*TNFRSF4* and *TNFRSF1A*), and genes involved in immune cell chemotaxis (*CCL5*), adhesion, and migration (*CD44*). Moreover, cell migration was also overrepresented in the turquoise module and included the expression of different integrins (*ITGB5, ITGB4, ITGB3, ITGB2*, and *ITGB7*) and other molecules involved in cell adhesion (e.g., *CD151* and *CD24*). In fact, in early postpartum dairy cows, *CD44*, together with *SPP1*, was found to be positively associated with AT macrophage infiltration on SAT (10). We also quantified the expression of IBA1, or allograft inflammatory factor 1, a marker of macrophages, which was also increased in SCK SAT compared to NK SAT. In dairy cows, the number of IBA1-positive macrophages within SAT increased by 93% 3 weeks postpartum compared with 10 days prepartum and was associated with increased body condition score loss (50). While we were not able to assess CD44 protein expression in our IHC analysis, these results are in agreement with SPP1 and IBA1 changes in our study and with the concept that ketosis enhanced AT inflammatory responses and macrophage infiltration (13).

Although little is known regarding the trafficking dynamics of immune cells other than macrophages in the AT during the transition period in dairy cows (63), through IHC, we observed an increased expression of CD20, a common B-cell marker, in SCK when compared to NK. This finding might indicate increased B-cell trafficking to both VAT and SAT of SCK cows in the early postpartum period. Interestingly, the turquoise module, associated with SCK SAT, was overrepresented by the negative regulation of B-cell receptors (e.g., *PLCL2* and *CD22*) and the negative regulation of interleukin-2 production signaling pathways. B-cell receptor signaling is a tightly regulated process, important to reduce impaired lymphocyte development and excessive lymphocyte activation (64). The negative regulation of interleukin-2 production signaling pathways might be an additional indication of B-cell activity regulation, since IL-2 promotes the proliferation of activated B cells and induces their differentiation into plasma cells (65, 66). Together, these findings suggest that although we observe greater trafficking of B cells to SAT of SCK cows, BPs associated with B-cell regulation are activated as well.

Analyzing the BPs associated with the green and turquoise modules, aligned with results for IHC, we observed evidence that the AT of SCK cows presents a pro-inflammatory profile, which might be associated with a dysregulation of lipid homeostasis and adipocyte sensitivity to insulin. Nevertheless, it is important to note crucial differences in the response to SCK between SAT and VAT when observing the activated and inactivated pathways evaluated by IPA. For example, on SAT, LXR/RXR was activated in SCK, while on VAT, this pathway was inactivated in SCK cows when compared to NK VAT. LXR/RXR are nuclear receptors involved in the regulation of lipid metabolism, immune system, and cholesterol catabolism. In terms of immune response, LXR/RXR activation results in anti-inflammatory activity, modulation of TLR signaling, and pro-resolving macrophage differentiation, among others (67). Furthermore, we observed that, while in VAT, SCK activated many pathways related to immune function and inflammation (e.g., pathogen-induced cytokine storm signaling, dendritic cell maturation, IL-8 signaling, and TREM1 signaling), these pathways were inactivated in SAT of SCK when compared to NK SAT. In a recent publication analyzing the oxylipin profile of the same AT samples utilized in the present study, we observed similar tendencies of SCK on dampening or shifting oxylipin metabolism as a way to regulate inflammation or preserve tissue homeostasis (68); while SAT demonstrated a more protective, anti-inflammatory or pro-resolving oxylipin profile, VAT was more metabolically active and sensitive to systemic perturbations associated with SCK. Overall, these results highlight the complexity of the AT response to SCK, and how different functional genomics approaches are essential to better comprehend these effects, especially considering the depot-specific responses.

In NK cows, the blue module was positively associated with NK VAT and pointed to many overrepresented BPs, indicating a successful adaptation to the postpartum NEB. For example, the fatty acid beta-oxidation BP, which included numerous genes regulating mitochondrial activity, such as *ACAA2, ACAT1*, and *CPT1C*, is a major metabolic pathway for lipid catabolism and energy synthesis essential during periods of increased lipid mobilization and high circulating NEFA (69, 70), such as the postpartum period in dairy cows. We also observed the overrepresentation of the response to oxidative stress BP, containing *MSRB2, NEIL1*, and *GCLM* genes. The production of reactive oxygen species (ROS) through increased mitochondrial respiration by non-phagocytic cells (e.g., fibroblast and endothelial cells) (71) is a response to intense lipolysis and macrophage infiltration during NEB (72). This, in turn, induces the activation of an antioxidant transcriptional network to reduce lipolysis-mediated oxidative stress in postpartum high-producing dairy cows (72). Another important BP overrepresented in NK VAT (blue module) was the fatty acid metabolic process, enriched for *GPAT4*, *PPARG*, and *PPARA* gene expression. The *PPARs*, a group of nuclear receptor proteins, are key regulators of energy production, lipid metabolism, and inflammation (73). *PPARG* is a master regulator of lipid biosynthesis, adipocyte differentiation, and insulin sensitivity (74), while *PPARA* promotes beta-oxidation during fasting states (75). In addition, *PPARG* regulates macrophage activity and inhibits the production of pro-inflammatory cytokines, while *PPARA* inhibits canonical NF-κB signaling (73, 76). Notably, *PPARG* and *PPARGC1A* were central activated upstream regulators in VAT *vs.* SAT of NK cows, thus implying that, like in humans (77), *PPARG* agonism might be a potential therapeutic target for metabolic disorders in dairy cows. A previous study utilizing non-pregnant, non-lactating dairy cows treated with intravenous 2,4-thiazolidinedione (TZD) injections for 2 weeks reported an upregulation of *PPARG* in SAT and increased blood concentrations of glucose, insulin, and BHB, while the concentration of NEFA and adiponectin remained unchanged (78). While the authors suggested the use of TZD during the transition period to improve insulin homeostasis and to prevent excessive body condition score losses, their effect on VAT, inflammatory status, and metabolic disease prevention was not addressed by the study, thus requiring further elucidation. Altogether, we observed a relation between NK VAT and an increase in mitochondrial activity, oxidative stress, and antioxidant response, as well as the enhancement of PPAR-mediated lipid and inflammatory homeostatic responses. These results might suggest mechanisms of metabolic adaptation necessary to successfully cope with NEB during postpartum and early lactation.

Lastly, the association of the brown module with NK SAT highlighted the histone H3-K36 demethylation as the most overrepresented BP and included the enrichment of *KDM4B.* The expression of *KDM4B* in AT is critical for the regulation of systemic metabolism via enhancing energy expenditure in adipocytes (79), as evidenced by its protective effect against obesity and metabolic dysfunction (80). Like the blue module (NK VAT), the fatty acid metabolic process was also overrepresented in the brown module, and included the expression of genes such as *CPT1B*, which regulates mitochondrial beta-oxidation, and *PPARD*, which induces fatty acid oxidation and energy dissipation, improving lipid profile and reducing adiposity in mice (81). Interestingly, corroborating with greater fatty acid beta-oxidation in NK VAT, the brown module was overrepresented by the mitochondrial respiratory chain complex I assembly BP, enriched for mitochondrial genes. Thus, in agreement with the observed findings in NK VAT, results for NK SAT suggest that independent of AT depot, the physiological adaptations to NEB during postpartum involve increased oxidation and mitochondrial activity in the AT. In summary, the BPs in the blue and brown modules, signatures from NK, reflect adjusted mitochondrial metabolism for the oxidization of lipids as a source of energy and set the stage for inflammation resolution and recovery of lipid metabolism homeostasis during NEB.

## Conclusions

Our results evidence depot-specific effects of SCK on the transcriptome and immunohistochemical profiles of flank SAT and omental VAT in postpartum dairy cows. Our findings demonstrate depot-specific mechanisms that may be involved in the pathogenesis of ketosis and in the successful transition to lactation, despite the NEB and increased lipid mobilization. Our findings suggest a more homeostatic role for SAT in cows with SCK, while VAT presents a pro-inflammatory profile, which might be related to dysregulation of lipid homeostasis and insulin sensitivity, likely contributing, to a higher extent, to the pathogenesis of metabolic disorders. Our analyses indicate the need to evaluate AT-related disorders in a depot-specific manner. Further studies are warranted for the development of targeted preventive and therapeutic strategies for SCK of high-producing dairy cows.

## Data Availability

Data is available at the Gene Expression Omnibus Repository (GEO; GSE245350).

## Supplementary material

Supplementary Figure 1 Immunohistochemistry controls for CD3, SPP1, CD20 and IBA1 antibodies.

Supplementary Table 1 Full list of differentially expressed genes (DEGs) in subcutaneous (SAT) and visceral adipose tissue (VAT) of cows with (SCK) and without (NK) subclinical ketosis identified by GeTMM analysis. Comparisons include: SCK *vs.* NK, SCK SAT *vs.* NK SAT, SCK VAT *vs.* NK VAT, SCK VAT *vs.* SCK SAT, NK VAT *vs.* NK SAT, and VAT *vs.* SAT.

Supplementary Table 2 Identified exclusively expressed genes (DEGs) in a depot- and disease-specific manner.

Supplementary Table 3 Weighted gene co-expression network analysis (WGCNA) and genes with the highest intramodular connectivity (hub genes, MM ≥ 0.90; denoted by bold font) in each identified module.

Supplementary Table 4 Overrepresented biological processes (BP) in each module identified by WGCNA analysis performed on fold enrichment values with *p value* < 0.01 (represented in bold).

Supplementary Table 5 Ingenuity Pathway Analysis of activated and inactivated pathways in subcutaneous (SAT) and visceral adipose tissue (VAT) of cows with (SCK) and without (NK) subclinical ketosis. Comparisons include: SCK *vs.* NK, SCK SAT *vs.* NK SAT, SCK VAT *vs.* NK VAT, SCK VAT *vs.* SCK SAT, NK VAT *vs.* NK SAT, and VAT *vs.* SAT.

Supplementary Table 6 Potential upstream regulators of genes differentially expressed between SCK SAT *vs.* NK SAT, SCK VAT *vs.* NK VAT, SCK VAT *vs.* SCK SAT and NK VAT *vs.* NK SAT, SCK *vs.* NK, and VAT *vs.* SAT. Upstream regulators were identified by analysis of the DEGs using Ingenuity Pathway Analysis (IPA).

## References

[1] Overton TR Waldron MR. Nutritional management of transition dairy cows: stratagies to optimize metabolic health. J Dairy Cows. (2004) 87:E105–E19. doi: 10.3168/jds.S0022-0302(04)70066-1

[2] Grummer RR. Impact of changes in organic nutrient metabolism on feeding the transition dairy cow. J Anim Sci. (1995) 73:2820–33. doi: 10.2527/1995.7392820x 8582873

[3] Drackley JK. Biology of dairy cows during the transition period: the final frontier? J Dairy Sci. (1999) 82:2259–73. doi: 10.3168/jds.S0022-0302(99)75474-3 10575597

[4] Esposito G Irons PC Webb EC Chapwanya A. Interactions between negative energy balance, metabolic diseases, uterine health and immune response in transition dairy cows. Anim Reprod Sci. (2014) 144:60–71. doi: 10.1016/j.anireprosci.2013.11.007 24378117

[5] Lopreiato V Mezzetti M Cattaneo L Ferronato G Minuti A Trevisi E. Role of nutraceuticals during the transition period of dairy cows: A review. J Anim Sci Biotechnol. (2020) 11:1–18. doi: 10.1186/s40104-020-00501-x 32864127 PMC7450574

[6] Bauman DE Currie WB. Partitioning of nutrients during pregnancy and lactation: A review of mechanisms involving homeostasis and homeorhesis. J Dairy Sci. (1980) 63:1514–29. doi: 10.3168/jds.S0022-0302(80)83111-0 7000867

[7] De Koster J Nelli RK Strieder-Barboza C de Souza J Lock AL Contreras GA. The contribution of hormone sensitive lipase to adipose tissue lipolysis and its regulation by insulin in periparturient dairy cows. Sci Rep. (2018) 8:1–11. doi: 10.1038/s41598-018-31582-4 30190510 PMC6127149

[8] Contreras GA Strieder-Barboza C Raphael W. Adipose tissue lipolysis and remodeling during the transition period of dairy cows. J Anim Sci Biotechnol. (2017) 8:41. doi: 10.1186/s40104-017-0174-4 28484594 PMC5420123

[9] De Koster J Nelli RK Strieder-Barboza C de Souza J Lock AL Contreras GA. The contribution ofxx xxhormone xxsensitive xxlipase to adipose tissue lipolysis and its regulation by insulin in periparturient dairy cows. Sci Rep. (2018) 8:13378. doi: 10.1038/s41598-018-31582-4 30190510 PMC6127149

[10] De Koster J Strieder-Barboza C de Souza J Lock AL Contreras GA. Short communication: effects of body fat mobilization on macrophage infiltration in adipose tissue of early lactation dairy cows. J Dairy Sci. (2018) 101:7608–13. doi: 10.3168/jds.2017-14318 29885887

[11] Strieder-Barboza C de Souza J Raphael W Lock AL Contreras GA. Fetuin-A: A negative acute-phase protein linked to adipose tissue function in periparturient dairy cows. J Dairy Sci. (2018) 101:2602–16. doi: 10.3168/jds.2017-13644 29274966

[12] Ospina P Nydam D Stokol T Overton T. Evaluation of nonesterified fatty acids and beta-hydroxybutyrate in transition dairy cattle in the northeastern United States: critical thresholds for prediction of clinical diseases. J Dairy Sci. (2010) 93:546–54. doi: 10.3168/jds.2009-2277 20105526

[13] Contreras GA Kabara E Brester J Neuder L Kiupel M. Macrophage infiltration in the omental and subcutaneous adipose tissues of dairy cows with displaced abomasum. J Dairy Sci. (2015) 98:6176–87. doi: 10.3168/jds.2015-9370 26117355

[14] Baird GD. Primary ketosis in the high-producing dairy cow: clinical and subclinical disorders, treatment, prevention, and outlook. J Dairy Sci. (1982) 65:1–10. doi: 10.3168/jds.S0022-0302(82)82146-2 7042782

[15] Gohary K Overton MW Von Massow M LeBlanc SJ Lissemore KD Duffield TF. The cost of a case of subclinical ketosis in Canadian dairy herds. Can Vet J. (2016) 57:728–32. PMC4904808 27429460

[16] Rodriguez-Jimenez S Haerr KJ Trevisi E Loor JJ Cardoso FC Osorio JS. Prepartal standing behavior as a parameter for early detection of postpartal subclinical ketosis associated with inflammation and liver function biomarkers in peripartal dairy cows. J Dairy Sci. (2018) 101:8224–35. doi: 10.3168/jds.2017-14254 29935824

[17] McArt JAA Nydam DV Oetzel GR. Epidemiology of subclinical ketosis in early lactation dairy cattle. J Dairy Sci. (2012) 95:5056–66. doi: 10.3168/jds.2012-5443 22916909

[18] Steeneveld W Amuta P van Soest FJS Jorritsma R Hogeveen H. Estimating the combined costs of clinical and subclinical ketosis in dairy cows. PloS One. (2020) 15:e0230448. doi: 10.1371/journal.pone.0230448 32255789 PMC7138322

[19] Guard C. The costs of common diseases of dairy cattle (Proceedings). (2008).

[20] Kenéz Á Tienken R Locher L Meyer U Rizk A Rehage J. Changes in lipid metabolism and β-adrenergic response of adipose tissues of periparturient dairy cows affected by an energy-dense diet and nicotinic acid supplementation. J Anim Sci. (2015) 93:4012–22. doi: 10.2527/jas.2014-8833 26440181

[21] Kenéz Á Ruda L Dänicke S Huber K. Insulin signaling and insulin response in subcutaneous and retroperitoneal adipose tissue in holstein cows during the periparturient period. J Dairy Sci. (2019) 102:11718–29. doi: 10.3168/jds.2019-16873 31563314

[22] Michelotti TC Kisby BR Flores LS Tegeler AP Fokar M Crasto C. Single-nuclei analysis reveals depot-specific transcriptional heterogeneity and depot-specific cell types in adipose tissue of dairy cows. Front Cell Dev Biol. (2022) 10:1025240. doi: 10.3389/fcell.2022.1025240 36313560 PMC9616121

[23] Depreester E De Koster J Van Poucke M Hostens M Van Den Broeck W Peelman L. Influence of adipocyte size and adipose depot on the number of adipose tissue macrophages and the expression of adipokines in dairy cows at the end of pregnancy. J dairy Sci. (2018) 101:6542–55. doi: 10.3168/jds.2017-13777 29627241

[24] Sparks B Michelotti TC Tegeler AP Fiallo JF Flores LS Strieder-Barboza C. Targeted lipidomics reveals depot-specific effects of subclinical ketosis in adipose tissue oxylipid profile of dairy cows. In: 2023 Annual Meeting of American Dairy Science Association. Journal of Dairy Science, Ottawa, CAN (2023).

[25] Ferguson JD Galligan DT Thomsen N. Principal descriptors of body condition score in holstein cows. J Dairy Sci. (1994) 77:2695–703. doi: 10.3168/jds.S0022-0302(94)77212-X 7814740

[26] Ning M Zhao Y Li Z Cao J. Ketosis alters transcriptional adaptations of subcutaneous white adipose tissue in holstein cows during the transition period. Animals. (2022) 12:2238. doi: 10.3390/ani12172238 36077956 PMC9454750

[27] Ford HR Mitchell TM Scull T Benitez OJ Strieder-Barboza C. The effect of subclinical ketosis on the peripheral blood mononuclear cell inflammatory response and its crosstalk with depot-specific preadipocyte function in dairy cows. Animals. (2024) 14:1995. doi: 10.3390/ani14131995 38998107 PMC11240650

[28] Dobin A Davis CA Schlesinger F Drenkow J Zaleski C Jha S. Star: ultrafast universal rna-seq aligner. Bioinformatics. (2012) 29:15–21. doi: 10.1093/bioinformatics/bts635 23104886 PMC3530905

[29] Smid M Coebergh van den Braak RRJ van de Werken HJG van Riet J van Galen A de Weerd V. Gene length corrected trimmed mean of M-values (Getmm) processing of rna-seq data performs similarly in intersample analyses while improving intrasample comparisons. BMC Bioinf. (2018) 19:236. doi: 10.1186/s12859-018-2246-7 PMC6013957 29929481

[30] Langfelder P Horvath S. Wgcna: an R package for weighted correlation network analysis. BMC Bioinf. (2008) 9:559. doi: 10.1186/1471-2105-9-559 PMC2631488 19114008

[31] Davies CL Patir A McColl BW. Myeloid cell and transcriptome signatures associated with inflammation resolution in a model of self-limiting acute brain inflammation. Front Immunol. (2019) 10:1048. doi: 10.3389/fimmu.2019.01048 31156629 PMC6533855

[32] Li J Zhou D Qiu W Shi Y Yang J-J Chen S. Application of weighted gene co-expression network analysis for data from paired design. Sci Rep. (2018) 8:622. doi: 10.1038/s41598-017-18705-z 29330528 PMC5766625

[33] Abedi M Gheisari Y. Nodes with high centrality in protein interaction networks are responsible for driving signaling pathways in diabetic nephropathy. PeerJ. (2015) 3:e1284. doi: 10.7717/peerj.1284 26557424 PMC4636410

[34] Huang DW Sherman BT Lempicki RA. Systematic and integrative analysis of large gene lists using david bioinformatics resources. Nat Protoc. (2009) 4:44–57. doi: 10.1038/nprot.2008.211 19131956

[35] Dalmer TRA Clugston RD. Gene ontology enrichment analysis of congenital diaphragmatic hernia-associated genes. Pediatr Res. (2019) 85:13–9. doi: 10.1038/s41390-018-0192-8 PMC6760551 30287891

[36] Menarim BC El-Sheikh Ali H Loux SC Scoggin KE Kalbfleisch TS MacLeod JN. Transcriptional and histochemical signatures of bone marrow mononuclear cell-mediated resolution of synovitis. Front Immunol. (2021) 12:734322. doi: 10.3389/fimmu.2021.734322 34956173 PMC8692379

[37] Krämer A Green J Pollard JJR Tugendreich S. Causal analysis approaches in ingenuity pathway analysis. Bioinformatics. (2013) 30:523–30. doi: 10.1093/bioinformatics/btt703 PMC3928520 24336805

[38] Jensen LJ Kuhn M Stark M Chaffron S Creevey C Muller J. String 8—a global view on proteins and their functional interactions in 630 organisms. Nucleic Acids Res. (2008) 37:D412–D6. doi: 10.1093/nar/gkn760 PMC2686466 18940858

[39] Bertram CA Klopfleisch R. The pathologist 2.0: an update on digital pathology in veterinary medicine. Vet Pathol. (2017) 54:756–66. doi: 10.1177/0300985817709888 28578626

[40] Jones-Hall YL Skelton JM Adams LG. Implementing digital pathology into veterinary academics and research. J Vet Med Educ. (2022) 49:547–55. doi: 10.3138/jvme-2021-0068 34460355

[41] Riber-Hansen R Vainer B Steiniche T. Digital image analysis: A review of reproducibility, stability and basic requirements for optimal results. Apmis. (2012) 120:276–89. doi: 10.1111/j.1600-0463.2011.02854.x 22429210

[42] Kozlova NI Morozevich GE Gevorkian NM Berman AE. Implication of integrins α3β1 and α5β1 in invasion and anoikis of sk-mel-147 human melanoma cells: non-canonical functions of protein kinase akt. Aging (Albany NY). (2020) 12:24345. doi: 10.18632/aging.202243 33260159 PMC7762463

[43] Xu L Yu W Xiao H Lin K. Birc5 is a prognostic biomarker associated with tumor immune cell infiltration. Sci Rep. (2021) 11:390. doi: 10.1038/s41598-020-79736-7 33431968 PMC7801710

[44] Zhang Z Chen H Pan C Li R Zhao W Song T. Sulforaphane reduces adipose tissue fibrosis via promoting M2 macrophages polarization in hfd fed-mice. Biochim Biophys Acta (BBA) - Mol Cell Res. (2024) 1871:119626. doi: 10.1016/j.bbamcr.2023.119626 37977492

[45] Jönsson F Gurniak CB Fleischer B Kirfel G Witke W. Immunological responses and actin dynamics in macrophages are controlled by N-cofilin but are independent from adf. PloS One. (2012) 7:e36034. doi: 10.1371/journal.pone.0036034 22558315 PMC3338623

[46] Kamnev A Lacouture C Fusaro M Dupré L. Molecular tuning of actin dynamics in leukocyte migration as revealed by immune-related actinopathies. Front Immunol. (2021) 12:750537. doi: 10.3389/fimmu.2021.750537 34867982 PMC8634686

[47] Zeng L Xiao Q Chen M Margariti A Martin D Ivetic A. Vascular endothelial cell growth-activated xbp1 splicing in endothelial cells is crucial for angiogenesis. Circulation. (2013) 127:1712–22. doi: 10.1161/circulationaha.112.001337 23529610

[48] Reimold AM Iwakoshi NN Manis J Vallabhajosyula P Szomolanyi-Tsuda E Gravallese EM. Plasma cell differentiation requires the transcription factor xbp-1. Nature. (2001) 412:300–7. doi: 10.1038/35085509 11460154

[49] Poole DH Ndiaye K Pate JL. Expression and regulation of secreted phosphoprotein 1 in the bovine corpus luteum and effects on T lymphocyte chemotaxis. Reproduction. (2013) 146:527–37. doi: 10.1530/rep-13-0190 24019509

[50] Newman AW Miller A Leal Yepes FA Bitsko E Nydam D Mann S. The effect of the transition period and postpartum body weight loss on macrophage infiltrates in bovine subcutaneous adipose tissue. J Dairy Sci. (2019) 102:1693–701. doi: 10.3168/jds.2018-15362 30471901

[51] Soares RAN Vargas G Muniz MMM Soares MAM Cánovas A Schenkel F. Differential gene expression in dairy cows under negative energy balance and ketosis: A systematic review and meta-analysis. J Dairy Sci. (2021) 104:602–15. doi: 10.3168/jds.2020-18883 33189279

[52] Ji P Drackley JK Khan MJ Loor JJ. Inflammation- and lipid metabolism-related gene network expression in visceral and subcutaneous adipose depots of holstein cows. J Dairy Sci. (2014) 97:3441–8. doi: 10.3168/jds.2013-7296 24704230

[53] Ruda L Raschka C Huber K Tienken R Meyer U Dänicke S. Gain and loss of subcutaneous and abdominal fat depot mass from late pregnancy to 100 days in milk in german holsteins. J Dairy Res. (2019) 86:296–302. doi: 10.1017/S0022029919000542 31409432

[54] Häussler S Sadri H Ghaffari MH Sauerwein H. Symposium review: adipose tissue endocrinology in the periparturient period of dairy cows. J Dairy Sci. (2022) 105:3648–69. doi: 10.3168/jds.2021-21220 35181138

[55] Mann S. Symposium review: the role of adipose tissue in transition dairy cows: current knowledge and future opportunities. J Dairy Sci. (2022) 105:3687–701. doi: 10.3168/jds.2021-21215 34998568

[56] Novo LC Cavani L Pinedo P Melendez P Peñagaricano F. Genomic analysis of visceral fat accumulation in holstein cows. Front Genet. (2022) 12:803216. doi: 10.3389/fgene.2021.803216 35058972 PMC8764383

[57] Moreno-Viedma V Tardelli M Zeyda M Sibilia M Burks JD Stulnig TM. Osteopontin-deficient progenitor cells display enhanced differentiation to adipocytes. Obes Res Clin Pract. (2018) 12:277–85. doi: 10.1016/j.orcp.2018.02.006 29519755

[58] Wærp HKL Waters SM McCabe MS Cormican P Salte R. Long-term effects of prior diets, dietary transition and pregnancy on adipose gene expression in dairy heifers. PloS One. (2019) 14:e0218723. doi: 10.1371/journal.pone.0218723 31269511 PMC6609222

[59] Famulla S Lamers D Hartwig S Passlack W Horrighs A Cramer A. Pigment epithelium-derived factor (Pedf) is one of the most abundant proteins secreted by human adipocytes and induces insulin resistance and inflammatory signaling in muscle and fat cells. Int J Obes. (2011) 35:762–72. doi: 10.1038/ijo.2010.212 20938440

[60] Huang K-T Lin C-C Tsai M-C Chen K-D Chiu K-W. Pigment epithelium-derived factor in lipid metabolic disorders. Biomed J. (2018) 41:102–8. doi: 10.1016/j.bj.2018.02.004 PMC6138776 29866598

[61] An YA Crewe C Asterholm IW Sun K Chen S Zhang F. Dysregulation of amyloid precursor protein impairs adipose tissue mitochondrial function and promotes obesity. Nat Metab. (2019) 1:1243–57. doi: 10.1038/s42255-019-0149-1 PMC6980705 31984308

[62] Cao S Pan Y Tang J Terker AS Arroyo Ornelas JP Jin GN. Egfr-mediated activation of adipose tissue macrophages promotes obesity and insulin resistance. Nat Commun. (2022) 13:4684. doi: 10.1038/s41467-022-32348-3 35948530 PMC9365849

[63] Contreras GA Strieder-Barboza C De Koster J. Symposium review: modulating adipose tissue lipolysis and remodeling to improve immune function during the transition period and early lactation of dairy cows. J Dairy Sci. (2018) 101:2737–52. doi: 10.3168/jds.2017-13340 29102145

[64] Takenaka K Fukami K Otsuki M Nakamura Y Kataoka Y Wada M. Role of phospholipase C-L2, a novel phospholipase C-like protein that lacks lipase activity, in B-cell receptor signaling. Mol Cell Biol. (2003) 23:7329–38. doi: 10.1128/mcb.23.20.7329-7338.2003 PMC230318 14517301

[65] Hipp N Symington H Pastoret C Caron G Monvoisin C Tarte K. Il-2 imprints human naive B cell fate towards plasma cell through erk/elk1-mediated bach2 repression. Nat Commun. (2017) 8:1443. doi: 10.1038/s41467-017-01475-7 29129929 PMC5682283

[66] Mingari MC Gerosa F Carra G Accolla RS Moretta A Zubler RH. Human interleukin-2 promotes proliferation of activated B cells via surface receptors similar to those of activated T cells. Nature. (1984) 312:641–3. doi: 10.1038/312641a0 6438535

[67] Bilotta MT Petillo S Santoni A Cippitelli M. Liver X receptors: regulators of cholesterol metabolism, inflammation, autoimmunity, and cancer. Front Immunol. (2020) 11:584303. doi: 10.3389/fimmu.2020.584303 33224146 PMC7670053

[68] Sparks BB Ford H Michelotti TC Strieder-Barboza C. Adipose tissue oxylipin profile changes with subclinical ketosis and depot in postpartum dairy cows. J Dairy Sci. (2025) 108:781–91. doi: 10.3168/jds.2024-25178 39343228

[69] Bionaz M Vargas-Bello-Pérez E Busato S. Advances in fatty acids nutrition in dairy cows: from gut to cells and effects on performance. J Anim Sci Biotechnol. (2020) 11:110. doi: 10.1186/s40104-020-00512-8 33292523 PMC7667790

[70] Busato S Bionaz M. The interplay between non-esterified fatty acids and bovine peroxisome proliferator-activated receptors: results of an *in vitro* hybrid approach. J Anim Sci Biotechnol. (2020) 11:91. doi: 10.1186/s40104-020-00481-y 32793344 PMC7419192

[71] Abuelo A Hernandez J Benedito JL Castillo C. Redox biology in transition periods of dairy cattle: role in the health of periparturient and neonatal animals. Antioxidants (Basel). (2019) 8(1):20. doi: 10.3390/antiox8010020 30642108 PMC6356809

[72] Salcedo-Tacuma D Parales-Giron J Prom C Chirivi M Laguna J Lock AL. Transcriptomic profiling of adipose tissue inflammation, remodeling, and lipid metabolism in periparturient dairy cows (Bos taurus). BMC Genomics. (2020) 21:824. doi: 10.1186/s12864-020-07235-0 33228532 PMC7686742

[73] Decara J Rivera P López-Gambero AJ Serrano A Pavón FJ Baixeras E. Peroxisome proliferator-activated receptors: experimental targeting for the treatment of inflammatory bowel diseases. Front Pharmacol. (2020) 11:730. doi: 10.3389/fphar.2020.00730 32536865 PMC7266982

[74] SChadinger SE Bucher NL Schreiber BM Farmer SR. Ppargamma2 regulates lipogenesis and lipid accumulation in steatotic hepatocytes. Am J Physiology-Endocrinology Metab. (2005) 288:E1195–205. doi: 10.1152/ajpendo.00513.2004 15644454

[75] Pawlak M Lefebvre P Staels B. Molecular mechanism of pparα Action and its impact on lipid metabolism, inflammation and fibrosis in non-alcoholic fatty liver disease. J Hepatol. (2015) 62:720–33. doi: 10.1016/j.jhep.2014.10.039 25450203

[76] Pereira AC Oliveira R Castro AC Fernandes R. Does pro(12)Ala polymorphism enhance the physiological role of pparγ2? PPAR Res. (2013) 2013:401274. doi: 10.1155/2013/401274 23983677 PMC3747383

[77] Berger JP Akiyama TE Meinke PT. Ppars: therapeutic targets for metabolic disease. Trends Pharmacol Sci. (2005) 26:244–51. doi: 10.1016/j.tips.2005.03.003 15860371

[78] Hosseini A Tariq MR Trindade da Rosa F Kesser J Iqbal Z Mora O. Insulin sensitivity in adipose and skeletal muscle tissue of dairy cows in response to dietary energy level and 2,4-thiazolidinedione (Tzd). PloS One. (2015) 10:e0142633. doi: 10.1371/journal.pone.0142633 26571137 PMC4646636

[79] Kang C Saso K Ota K Kawazu M Ueda T Okada H. Jmjd2b/kdm4b inactivation in adipose tissues accelerates obesity and systemic metabolic abnormalities. Genes Cells. (2018) 23:767–77. doi: 10.1111/gtc.12627 30073721

[80] Cheng Y Yuan Q Vergnes L Rong X Youn JY Li J. Kdm4b protects against obesity and metabolic dysfunction. Proc Natl Acad Sci U.S.A. (2018) 115:E5566–e75. doi: 10.1073/pnas.1721814115 PMC6004484 29844188

[81] Wang Y-X Lee C-H Tiep S Yu RT Ham J Kang H. Peroxisome-proliferator-activated receptor δ Activates fat metabolism to prevent obesity. Cell. (2003) 113:159–70. doi: 10.1016/S0092-8674(03)00269-1 12705865

