# Supplementary figures and images for: Subclinical ketosis in postpartum dairy cows alters the adipose tissue immunological profile in a depot-specific manner
Source: Front Immunol. 2025 Jun 17;16:1578669. doi: 10.3389/fimmu.2025.1578669 (PMC12212114; doi:10.3389/fimmu.2025.1578669)

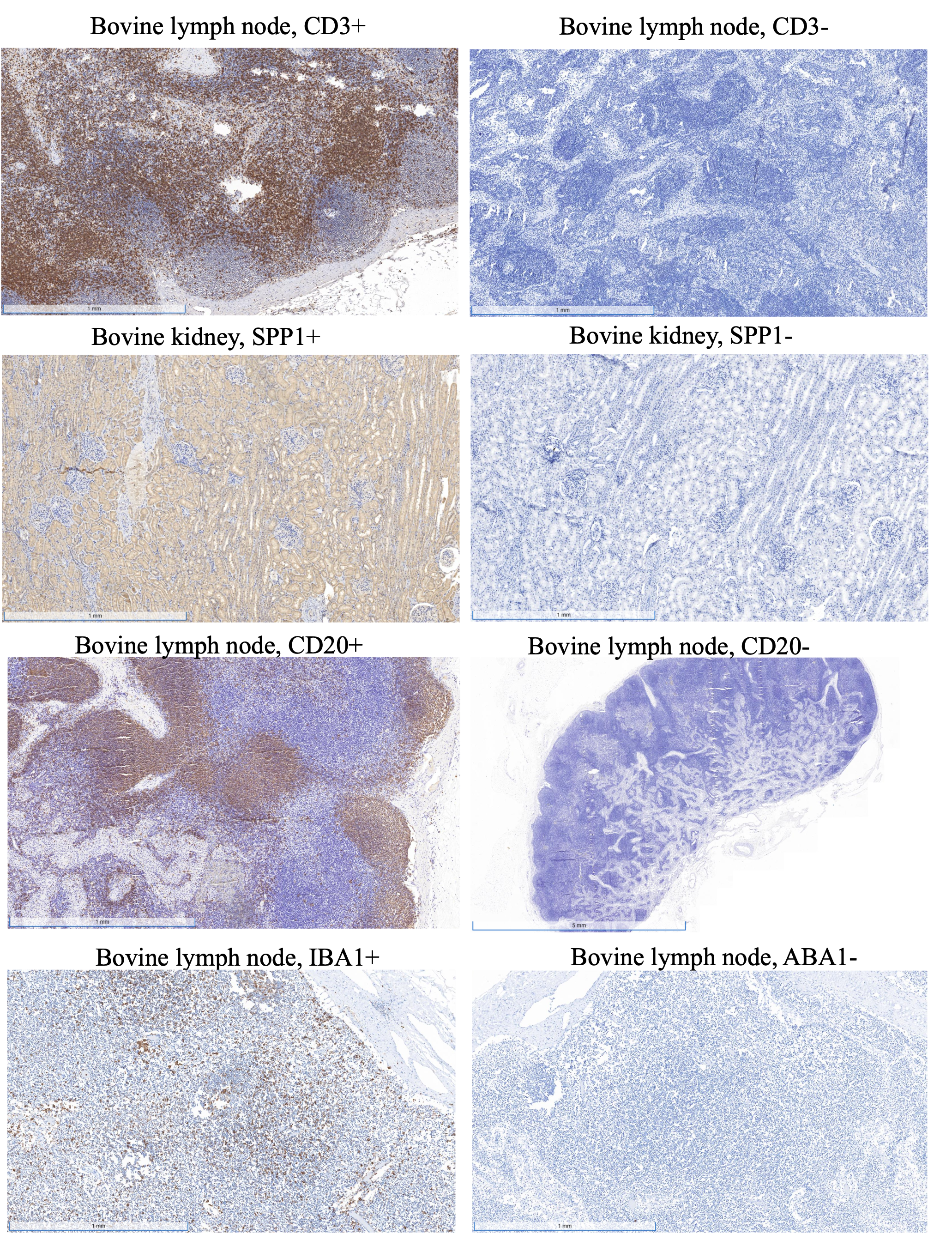

Supplement: Supplementary Figure 1 — Immunohistochemistry controls for CD3, SPP1, CD20 and IBA1 antibodies. [file Image1.png]
